# The Double Burden of Malnutrition and Associated Factors among South Asian Adolescents: Findings from the Global School-Based Student Health Survey

**DOI:** 10.3390/nu13082867

**Published:** 2021-08-20

**Authors:** Sara Estecha Querol, Romaina Iqbal, Laura Kudrna, Lena Al-Khudairy, Paramijit Gill

**Affiliations:** 1 Warwick Centre for Global Health, Warwick Medical School, University of Warwick, Coventry CV4 7AL, UK; p.gill.1@warwick.ac.uk; 2 Division of Health Sciences, Warwick Medical School, University of Warwick, Coventry CV4 7AL, UK; lena.al-khudairy@warwick.ac.uk; 3 Department of Community Health Sciences, Aga Khan University, Karachi 74800, Pakistan; romaina.iqbal@aku.edu; 4 Institute of Applied Health Research, University of Birmingham, Birmingham B15 2TT, UK; l.kudrna@bham.ac.uk

**Keywords:** adolescents, South Asia, malnutrition, stunting, thinness, overweight, GSHS

## Abstract

The health and nutrition of the global adolescent population have been under-researched, in spite of its significant size (1.2 billion). This study investigates the prevalence and associated factors of malnutrition (stunting, thinness and overweight) among adolescents living in South Asia. The sample analysed was 24,053 South Asian schooled adolescents aged 12–15 years that participated in the cross-sectional Global School-Based Student Health Survey (GSHS) between 2009 and 2016. The prevalence of stunting, thinness and overweight was calculated using the World Health Organization (WHO) Child Growth Reference 2007. Associations between the three forms of malnutrition and their possible associated factors were assessed with binary logistic regression analysis using bootstrapping as a resampling method. The overall prevalence of stunting in South Asia was 13%, thinness was 10.8% and overweight was 10.8%. In the logistic regression model of the overall pooled sample, the factors associated with adolescent malnutrition were: age, hygiene behaviours, social support, sedentary behaviour, and tobacco use. A substantial proportion of stunting, thinness and overweight was found among school-going South Asian adolescents, indicating that the double burden of malnutrition is present in this population. Future research should seek to further understand the relationship between all forms of malnutrition and its associated factors in the adolescent population.

## 1. Introduction

The growing challenge of populations experiencing multiple forms of malnutrition has drawn special attention from the global health community in the last three decades [1]. This concept, called the double burden of malnutrition, refers to the coexistence of overnutrition (overweight and obesity) and undernutrition (micronutrient deficiencies, underweight, and childhood stunting and wasting) at country, household, and individual levels [2]. This health issue is especially prevalent and persistent in low and middle-income countries (LMIC) [3]. The Lancet’s Series on the Double Burden of Malnutrition [2,4,5,6] reports that one in three LMIC are affected by the double burden of malnutrition. As a result of the global nutrition transition, larger numbers of people are expected to experience it across the life-course, typically suffering from undernutrition in childhood to overnutrition in adulthood [6].

Early life undernutrition may result in increased odds of childhood mortality, reduced capacity for physical work, lower school performance, and greater susceptibility to infectious diseases [7]. Adult overweight/obesity has been extensively associated with higher risk of non-communicable diseases such as hypertension, diabetes, cancer, stroke, and ischemic heart disease [7]. These diverse forms of ill health contribute to lower wages, loss of productivity and higher medical costs [5]. Thus, the double burden of malnutrition introduces considerable global health challenges that need to be urgently addressed [4]. The 2030 Agenda for Sustainable Development [8] and the United Nations Decade of Action on Nutrition 2016–2025 [9] aim to mobilise action and accelerate progress towards ending malnutrition in all its forms. Double-duty actions seek to tackle both forms of malnutrition simultaneously [10], and therefore have the potential to improve nutrition globally and promote health at all life stages across LMIC [4].

Early stages in life are critical windows for addressing nutritional burden and preventing the consequences of intergenerational malnutrition [11]. Adolescence presents a key opportunity for catching up with growth and habit formation, shaping health and eating behaviours that persist into adulthood [12]. Yet, the double burden of malnutrition among adolescents living in LMIC remains insufficiently investigated [13].

In the South Asian context, the prevalence of undernutrition in children and adolescents remains considerably high compared to other regions, despite a decline over the past three decades [14,15,16]. In addition, South Asia is currently facing the challenge of overnutrition which is exacerbating the double burden of malnutrition [2]. Recent epidemiological studies on the double burden of malnutrition in South Asia have focused on women of reproductive age [17,18,19,20,21,22,23] and mother-child pairs [24,25,26]. However, only a few studies quantified the double burden of malnutrition among adolescents in India, overlooking the rest of the South Asian countries [27,28,29,30]. These publications identified factors contributing to malnutrition such as socioeconomic status [27,28,29,30], hygiene behaviour [27], maternal education [29,30] and urban/rural residence [29].

A more comprehensive picture of the double burden of malnutrition in all South Asian countries is essential to improve adolescent health and nutritional status in this context. This study aims to address these gaps in knowledge by examining country and regional prevalence of the burden of stunting, thinness and overweight (adolescent malnutrition indicators) among South Asian adolescents aged 12–15 years. Additionally, this study investigates malnutrition associated factors and their association with these three forms of malnutrition.

## 2. Materials and Methods

### 2.1. Study Design and Participants

The Global School-based student Health Survey (GSHS) is a cross-sectional school-based survey conducted among students living in LMIC that uses standardised procedures to assess health behaviours and protective factors such as alcohol use, dietary behaviours, drug use, hygiene, mental health, physical activity, other protective factors, sexual behaviours, tobacco use, and violence and unintentional injury. The GSHS samples are representative of all students in a given country. Further details on the GSHS are published elsewhere [31]. We selected surveys from the eight South Asian countries where participants were asked about more health behaviours and protective factors and where the response rate was higher than 70%. Hence, the surveys included in this study were Pakistan 2009, Afghanistan 2014, Bangladesh 2014, India 2007, Maldives 2009, Nepal 2015, Sri Lanka 2016, and Bhutan 2016. We limited the analysis to well-defined age categories (‘12’, ‘13’, ‘14’, and ‘15 years old’) and excluded adolescents from ‘11 years or younger’ and ‘16 years or older’ categories because the exact age of the individual was required to calculate adolescent malnutrition indicators. Participants with missing information (age, sex, weight or height) or biologically implausible values (defined by WHO as extreme z-scores for weight, height and BMI [32]) were excluded from this analysis.

GSHS surveys employ a two-stage sample design where the first sampling units are clusters and the second sampling units are elements of those clusters. More specifically, the first level of the GSHS sample selection process was schools, that were independently selected within each country. In the second stage, classrooms were selected within each school, and a questionnaire was completed by each attending student [33]. The GSHS surveys are publicly available (https://www.who.int/teams/noncommunicable-diseases/surveillance/systems-tools/global-school-based-student-health-survey Accessed on 19 August 2021), and they were approved by the national government’s administrative body and the corresponding institutional ethics board review [31]. This study is a post-hoc analysis of the eight South Asian countries’ GSHS surveys, and as such, no ethical approval was required.

### 2.2. Study Variables

The GSHS uses core questionnaire modules, core-expanded questions, and country-specific questions that are combined to form a questionnaire that the students completed during one regular class period. Fifty-six health behaviours and protective factors (note that throughout this manuscript we will be referring to these variables as health behaviour variables) were collected, but not all of these variables are included in the South Asian surveys. In this analysis, variables were included if they were: (1) collected across all South Asian countries, and (2) relevant within existing established frameworks on adolescent nutritional status [11,14,34,35]. In total, 16 health behaviour variables (explanatory variables) were identified as potential predictors of adolescent malnutrition and included in the analyses. Dichotomous variables were created regardless of the number of responses to simplify the analyses and interpretation. Supplementary Material Table S1 provides detailed information on the definitions and coding of these variables.

Anthropometric measurements (height in cm and weight in kg) were collected by trained GSHS survey staff. Body mass index (BMI) was calculated as weight in kilograms divided by the square of height in meters. Adolescent malnutrition indicators (outcome variables) were assessed based on the WHO growth reference for school-aged children and adolescents [36] and the WHO macro was used to calculate z-scores [32]. Stunting was defined as height-for-age <2 SDs below the WHO Child Growth Reference median. Thinness was defined as BMI-for-age <2 SDs below the WHO Growth Reference median. Overweight/obesity (referred to as overweight in this manuscript) was defined as BMI-for-age >1 SD above the WHO Growth Reference median.

### 2.3. Statistical Analysis

To account for the GSHS two-stage sample design, this analysis applied the sampling weights, clustering, and stratification using the svy command in Stata v16 [37]. Prevalence of the three forms of malnutrition and health behaviour variables were calculated at the country level and South Asian level (pooled sample). We conducted a chi-square test of independence to examine the associations between sex and adolescent malnutrition indicators. In addition, the association between country and each health behaviour variable was evaluated using chi-squared tests. Statistical significance of the chi-square statistic was set at *p* < 0.05. Maps displaying prevalence of stunting, thinness and overweight among South Asian students were created using Tableau.

Bivariate analysis checked the independent association of each health behaviour variable to the outcome (*p* < 0.05, using chi-squared test). Collinearity was assessed to identify pairs of health behaviour variables that were correlated (r > 0.5, using Pearson’s correlation test). In addition, we computed other collinearity diagnostic measures (VIF > 10, tolerance value < 0.1 and condition index > 10) using collin command in Stata. None of the covariates were identified to be strongly collinear. Regression was used to examine the associations between malnutrition indicators and health behaviour variables. Binary logistic regression was applied to predict the outcome variable informed by a set of health behaviour variables—potential associated factors—calculating odds ratios (ORs). Manual backward stepwise regression was used to develop multivariable logistic regression models of the predictors of malnutrition in South Asian students aged 12–15 years. Only significant health behaviour variables (α < 0.05, using the Wald test) were retained in the reduced model, except for age, sex and country, which were considered traditional confounders. Full and reduced models (only including explanatory variables identified as significant using backward stepwise regression) of stunting, thinness and overweight at the South Asian and country level were examined. The bootstrap resampling method was used for a more accurate estimation of the standard errors in logistic regression.

## 3. Results

The characteristics of the GSHS datasets included in this study are shown in Table 1. The response rate ranged from 69% (Nepal) to 91% (Bangladesh). The sample size varied from 1493 participants in Afghanistan to 7327 in India. A total of 24,053 adolescents completed height, weight, sex and age measurements. The average age of students was 13.99 years (SD ± 0.89).

### 3.1. Prevalence of Stunting, Thinness and Overweight

**Figure 1:**
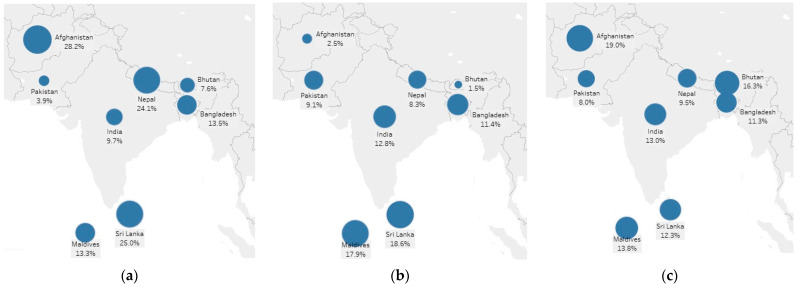
(**a**) Stunting (height-for-age < 2 SDs), (**b**) thinness (Body mass index (BMI)-for-age < 2 SDs), and (**c**) overweight (BMI-for-age > 1 SDs) prevalence of adolescents by South Asian country, GSHS data.

The overall prevalence of adolescent stunting in South Asia was 13%—this ranged from 3.9% in Pakistan to 28.2% in Afghanistan (χ^2^ = 1023.11, *p* < 0.001) (Figure 1a). Table 2 shows that stunting prevalence was 11.6% in boys and 14.8% in girls in the pooled sample (*p* = 0.06). There was no significant association between sex and stunting in any of the South Asian countries except from Pakistan where stunting in girls was more likely than in boys (*p* < 0.001).

The overall prevalence of adolescent thinness in South Asia was 10.8%—this ranged from 1.5% in Bhutan to 18.6% in Sri Lanka (χ^2^ = 159.5, *p* = 0.04) (Figure 1b). Table 3 shows that thinness prevalence was 11.8% in boys and 9.3% in girls in the pooled sample (*p* = 0.11). The proportion of students who were thin did not differ by sex in Pakistan, Afghanistan, Bangladesh, India, Maldives and Sri Lanka. However, there was a significant effect of sex on thinness in Nepal (*p* = 0.03), and Bhutan (*p* = 0.001) with boys being thinner than girls (*p* = 0.001).

The overall prevalence of adolescent overweight in South Asia was 10.8%—this ranged from 8% in Pakistan to 19% in Afghanistan (χ^2^ = 109.14, *p* = 0.2) (Figure 1c). Table 4 shows that overweight prevalence at the South Asian level was 11.4% in boys and 9.9% in girls (*p* = 0.3). The association between sex and overweight was not significant in Afghanistan, Bangladesh, Nepal, and Sri Lanka. In contrast, results indicated a significant difference between sexes in the remaining countries. Boys were more likely to be overweight in India (*p* = 0.01) and Maldives (*p* = 0.02), while girls were more likely to be overweight in Pakistan (*p* = 0.001) and Bhutan (*p* = 0.002).

### 3.2. Prevalence of Health Behaviours

Table 5 shows the prevalence of health behaviours at the country level and South Asian level. Around 14% of adolescents in the pooled sample consumed the recommended amount of 5 portions of fruits and vegetables per day [39]. Feeling frequently lonely or worried was reported by 10.1% and 5.9% of adolescents, respectively. The overall prevalence of tobacco use was 6.3%. Recommended levels of physical activity were met by 27.1% of adolescents in this sample [40]. Over half of the respondents (54%) brushed their teeth twice a day following American Dental Association recommendations [41]. Washing hands frequently was reported in more than three out of four adolescents. Feeling supported by parents was reported in around half of the sample. There was a significant association between country and all health behaviour variables (*p* < 0.05) apart from washing hands before meals and after toilet.

### 3.3. Factors Associated with Malnutrition Indicators

The bivariate analysis showed that anxiety (*p* = 0.03) and tobacco use (*p* = 0.04) were significantly associated with stunting in the pooled sample (Supplementary Material Table S2). Sedentary behaviour was significantly associated with thinness (*p* = 0.01) (Supplementary Material Table S3). The following factors were significantly associated with overweight (Supplementary Material Table S4): tobacco use (*p* = 0.001), sedentary behaviour (*p* = 0.009), and tooth brushing (*p* = 0.005).

The multivariable logistic regression reduced model of the pooled sample (Table 6) showed that age and country of residence were associated with stunting. Students aged 13, 14 and 15 years had more than two times the odds of being stunted than students aged 12 years (*p* < 0.001). Students living in Afghanistan (OR = 10.9, *p* < 0.001), Bangladesh (OR = 4.3, *p* < 0.001), India (OR = 2.7, *p* < 0.001), Maldives (OR = 3.2, *p* < 0.001), Nepal (OR = 9, *p* < 0.001), Sri Lanka (OR = 8.4, *p* < 0.001), and Bhutan (OR = 2.2, *p* < 0.001) exhibited a greater risk of being stunted than students living in Pakistan. Peer support was negatively associated with stunting (OR = 0.7, *p* < 0.001).

In the reduced model for thinness (Table 7), several factors were associated with this malnutrition indicator at the South Asian level: being 13 years old (OR = 1.6, *p* = 0.003) versus being 12 years old; living in Afghanistan (OR = 0.3, *p* < 0.001), India (OR = 1.7, *p* = 0.001), Maldives (OR = 3.1, *p* < 0.001), Sri Lanka (OR = 3, *p* < 0.001) and Bhutan (OR = 0.2, *p* < 0.001) versus living in Pakistan; sedentary behaviour (OR = 0.6, *p* = 0.001); and tooth brushing (OR = 0.7, *p* = 0.01).

In the reduced model for overweight (Table 8), South Asian schooled adolescents being 13 years (OR = 0.6, *p* = 0.006), 14 years (OR = 0.5, *p* = 0.001) and 15 years old (OR = 0.5, *p* = 0.001) were less likely to be overweight than their peers aged 12 years old. Students living in Afghanistan (OR = 2.6, *p* < 0.001), India (OR = 1.5, *p* = 0.02), Maldives (OR = 1.7, *p* = 0.03), Sri Lanka (OR = 1.6, *p* = 0.03), and Bhutan (OR = 2.2, *p* < 0.001) versus those living in Pakistan had significantly greater odds of being overweight than students living in Pakistan. Several factors were also associated with overweight: tobacco use (OR = 0.4, *p* = 0.001), tooth brushing (OR = 1.4, *p* = 0.003), washing hands with soap (OR = 1.6, *p* = 0.02), and parental involvement in school (OR = 0.8, *p* = 0.03).

In addition to the pooled sample models, full and reduced models of the three forms of malnutrition at country level were also examined. Factors associated with malnutrition indicators varied across countries and no clear trend was identified (Supplementary Material, Table S5–S28).

## 4. Discussion

In this secondary data analysis of the GSHS, we estimated the prevalence of the double burden of malnutrition among in-school adolescents in South Asian countries (Pakistan, Afghanistan, Bangladesh, India, Maldives, Nepal, Sri Lanka and Bhutan). This study showed that the overall prevalence of stunting in South Asia was 13%, thinness was 10.8% and overweight was 10.8%, with significant geographical variations in stunting and thinness. Sex was not associated with adolescent malnutrition indicators in the pooled sample; however, it was within some countries (stunting in Pakistan; thinness in Nepal and Bhutan; and overweight in Pakistan, India, Maldives, and Bhutan). Prevalence of health behaviours was calculated showing substantial differences across South Asian countries. We identified factors associated with malnutrition at the South Asian level: age, hygiene behaviours, social support, sedentary behaviour, and tobacco use.

Firstly, we calculated the prevalence of stunting, thinness and overweight in South Asia. The present findings suggest that adolescents in South Asia are affected by the double burden of malnutrition, consistent with global trends [13,29], and other studies from South Asia [27,42]. Pakistan showed the lowest rate for stunting (3.9%) and overweight (8%). Afghanistan presented the highest prevalence across South Asia of stunting (28.2%) and overweight (19%), contrasting with a low thinness rate (2.5%). Sri Lanka reported the highest rate for thinness (18.6%), co-existing with relatively high rates of stunting (25%) and overweight (13.2%). The lowest prevalence of thinness in South Asia was found in Bhutan (1.5%) as opposed to overweight prevalence (16.3%). It is important to point out that we could not report the prevalence of the double burden of malnutrition as high, medium or low in this study because malnutrition prevalence thresholds in adolescents have not yet been established. Since malnutrition thresholds of public health significance have been defined for children under 5 years of age [43], future research should aim to develop these thresholds for adolescent populations. Classifying the prevalence of stunting, thinness and overweight in adolescents may contribute to improving monitorization, identifying priorities, mobilising action and tailoring interventions addressing malnutrition.

This study demonstrates some significant associations between sex and adolescent malnutrition indicators. Pakistani girls were more likely to be stunted and overweight in comparison to boys. There was a significant relationship between malnutrition indicators and sex in Bhutan with boys being thinner than girls and girls being more overweight than boys. Findings also showed a significant effect for sex in India, Maldives and Nepal. Despite these associations in our sample, there is no distinct sex disparity trend. This is consistent with findings from previous reviews on underweight [44] and overweight [45] among South Asian adolescents. Although sex may not be a malnutrition risk factor, gender inequalities in health should be acknowledged in this population. Adolescent girls in South Asia are disproportionately affected by early marriage and pregnancy, sexual violence, reproductive morbidity and mortality, and deprived educational and employment opportunities; while adolescent boys suffer from injury, violent death, suicide, harmful drinking and tobacco smoking [46,47].

To our knowledge, no previous analyses have calculated adolescent malnutrition indicators at a country level in the eight South Asian countries. Our results are not directly comparable with previous publications [13,15,48,49,50]. It is problematic to compare regional malnutrition prevalence from previous studies [13,50] to our analyses because of different geographical classifications (WHO vs. World Bank classification). Although previous evidence [15,48,49] applied the same geographical classification, the age ranges of the target population differed. For instance, findings from NCD-RisC showed that more than a quarter of children and adolescents aged 5–19 were underweight and less than a tenth were overweight [15]. Nevertheless, this publication found that thinness prevalence in South Asia to be the highest worldwide. UNICEF reported that 11% of South Asian adolescent girls aged 15–19 years were stunted and 39% were thin [49]. Prevalence in children under 5 years of age living in South Asia was 30% for underweight, 37% for stunting and 4% for overweight [48]. This study adds to this information by reporting the overall prevalence of stunting, thinness and overweight among adolescents aged 12 to 15 years using a standardised methodology across the eight South Asian countries.

Secondly, the prevalence of health behaviours was examined in this study. We compared our results with other GSHS publications where possible (i.e., variables’ cut-offs are the same as ours). Only 14.3% of South Asian adolescents consumed the recommended fruit and vegetable intake (5/day). A study using GSHS datasets in 65 low-income and middle-income countries reported a higher percentage (25.7%) of adolescents who met the recommendations of daily fruit and vegetable intake [51]. Feeling lonely and anxious was reported by 10.1% and 5.9% of respondents in our pooled sample, respectively. Other GSHS studies found loneliness and anxiety to be 10.0% and 10.4% in Seychelles [52], and 7.5% and 6.2% in sub-Saharan Africa countries [53]. Psychological distress in this study was particularly outstanding among Afghan adolescents with 28.8% in loneliness and 21.84% in anxiety. It may be worth noting that at the time that the GSHS survey was conducted in Afghanistan, a war was impacting the country, which may help to explain the high percentages of in-school adolescents suffering from anxiety and loneliness. Previous literature found that Afghan adolescents suffer distress, trauma, violence, and war-related experiences affecting greatly their mental health [54].

We report a lower overall prevalence of tobacco use (5.3%) in comparison to previous GSHS publications in Seychelles (22%) [52], Thailand (14.1%) [55], African countries (10.2%) [56], and 68 low-income and middle-income countries (13.6%) [57]. Similarly, findings from the Global Youth Tobacco Survey showed that 5.4% of South Asian adolescents were cigarette smokers [58]. We hypothesise that relatively low tobacco use rates in South Asia are linked with strong social stigma and religious unacceptability which may reduce the smoking rate in this population. Additionally, these are subjective measures to reporting bias.

The prevalence of physically active adolescents at the South Asian level was 27.1% in this study. However, findings from the Africa region (16.6%), the Americas region (16.2%) and the Western Pacific region (13.8%) [59] indicated lower rates of physical activity. Half of the schooled adolescents in the present study reported active transportation such as walking or riding a bicycle, while adolescents in Africa (41.3%), America (40.4%), and Western Pacific (44.1%) regions reported lower rates of active transportation [59]. On the other hand, our study found a lower overall prevalence of sedentary behaviour (15.8%) compared to schooled adolescents living in Africa (41.3%), America (40.4%), and Western Pacific (44.1%) regions [59].

Results from the pooled sample showed that 54% of the adolescents reported brushing their teeth twice a day with large country variations ranging from 30.2% in Pakistan to 77.1% in Maldives. Pengpid and Peltzer (2014) found that daily brushing recommendations were met by 77.6% of adolescents from four Southeast Asian Countries [60]. The prevalence of frequent hand hygiene behaviour was lower in our study (washing hands before meals, after toilet use and with soap was 89.5%, 92.8%, and 79.9%, respectively) compared to another GSHS publication that analysed data on hand-washing practices across 80 countries (washing hands before meals, after toilet and with soap was 93.6, 94.4%, and 91.2%, respectively) [61].

The prevalence of students reporting that they had at least one close friend in our pooled sample was 91.9%, similar prevalence was reported in Latin America and the Caribbean (92%) [62] and in Africa (87.6%) [63]. Adolescents in our pooled sample reported a slightly higher rate of peer support (49.6%) compared to findings from Africa (39%) [63] and the Association of Southeast Asian Nations (ASEAN) Member States (40.4%) [64].

Half of the adolescents in this sample reported the presence of supportive parental figures (53% reported that their parents were involved in their academic activities, 53.4% reported that their parents understood their problems, and 50.3% reported that their parents monitor their leisure time activities). Maldives and Bhutan presented the lowest levels of parental support whereas the highest rates of perceived parental support were found in Sri Lanka. One in three adolescents in the Caribbean [65] reported that their parents often check their homework while less than half of adolescents reported parent involvement in school activities in a GSHS publication analysing data from 52 countries [66]. One in three adolescents had parents who understood their problems in sub-Saharan Africa [53], the Caribbean [65] and in another GSHS study from 52 countries [66]. Half of the adolescents from these two last studies [65,66] reported that their parents monitored their leisure time.

Thirdly, several health behaviours were found to be associated with adolescent malnutrition indicators (stunting, thinnenes and overweight) in multivariable logistic regression models of the pooled sample. We found that variables representing hygiene behaviours were associated with malnutrition. Tooth brushing was associated with two forms of malnutrition (thinness and overweight). In other South Asian studies, poor oral health predicted overweight [67,68,69] and underweight [68]. This study shows that handwashing practices were associated with nutritional status among adolescents living in South Asia. Likewise, inadequate handwashing practices had a significant association with poor nutritional status in adolescent girls living in eastern India [70].

Interestingly, this study did not find a significant association between sedentary behaviour and overweight. By contrast, previous research from Sri Lanka found association between sedentary behaviour and being overweight among school children [71]. Nevertheless, we found that sedentary behaviour was negatively associated with thinness. Sedentary behaviour implies a low energy expenditure which may lead to an increase in BMI, hence its protective effect against thinness.

In this analysis, tobacco use was found as a significant predictor for overweight. It is difficult to contextualise this finding because no studies were found on the association between smoking and nutritional status in South Asia, possibly due to a predominantly smokeless society and social taboos. Pengpid & Peltzer (2016) confirmed an association between tobacco use and overweight among girls in the AESAN member countries (Brunei, Cambodia, Indonesia, Laos, Malaysia, Myanmar, the Philippines, Singapore, Thailand, and Vietnam).

Peer support and parental involvement in school were associated with malnutrition at the South Asian level. Peer support was a protective factor against stunting while parental involvement in school protected against being overweight. Peers and parents have a great impact on adolescent food choices [72,73], especially on those who are overweight [74]. For example, Pengpid & Peltzer a reported positive association between peer support and overweight among school-going adolescents girls in the AESAN member countries [75] and a negative association between peer support and underweight [76]. Parental supervision was positively associated with underweight and parental bonding increased the odds for overweight or obesity among schooled adolescents in AESAN countries [76]. Although this is the first study that investigates the relationship between the double burden and peer influence in the South Asian context, these findings suggest that the double burden of malnutrition may be reduced with social support.

Fruits and vegetable consumption, psychological distress and physical activity have previously been associated with nutritional status, especially overweight, among South Asian adolescents [77,78,79]. However, these factors did not appear significant in the pooled sample. Additionally, traditional confounders such as sex and age were explored. The multivariable analysis did not provide evidence that sex was a risk factor for malnutrition at the South Asian level. This coincides with recent reviews that reported [44,45] an inconsistent association between nutritional status and sex within the literature. Older age was associated with a higher stunting probability. Previous evidence from India also found that the risk of stunting in late adolescence was higher than in early adolescence [80,81]. On the other hand, age was negatively associated with overweight in our study.

### Strengths and Limitations

The use of the GSHS is both a strength and a limitation. Regarding the strengths of the GSHS, there are no other global quality health surveys on South Asian adolescents up to date in which data is nationally representative and methodology is standardised across countries. This survey collects height, weight and BMI that are anthropometric measurements widely used to calculate malnutrition indicators and monitor prevalence at the population level. In terms of limitations, the cross-sectional, non-interventional design limits any causal interpretation, although the observed prevalence and associations could be used to generate hypotheses. Second, data on certain aspects of adolescents’ circumstances were not available in the GSHS, such as household socioeconomic status, ethnicity, religion, and rural/urban setting, so adjusting and stratifying the analysis to explore further inequalities among these subgroups was not possible. Third, GSHS does not collect nutritional biomarkers data, thus micronutrient deficiency as a form of malnutrition was not included in this analysis. The exclusion of micronutrient deficiencies from the malnutrition burden leads to underrate its impact on adolescent health and nutrition [82]. Fourth, the breadth of GSHS has been questioned due to its restriction to adolescents who are attending school as rates of absenteeism are high in LMIC [12]. The estimates of the double burden of malnutrition in this analysis apply only to adolescents who attend school, therefore findings cannot be generalised to all adolescent populations living in South Asia. Fifth, the reliability and validity of the GSHS questionnaire adapted for South Asian countries have not been investigated yet; however, two studies on schooled adolescents from Fiji [83] and Iran [84] found acceptable reliability and validity concluding that the questionnaire can serve as a reliable instrument for obtaining data on adolescent health behaviours.

This particular analysis also has limitations. Some important variables such as frequency of hunger, carbonated soft drink consumption and frequency of eating at fast-food restaurants were not included in this analysis due to the inclusion criteria, that is, only explanatory variables collected in the eight South Asian countries. Survey data were collected between 2008 and 2016, so comparisons between South Asian countries should be made with caution. Despite these limitations, this study is the first, to our knowledge, to examine the relationship between the double burden of malnutrition and associated factors among South Asian adolescents using a large pool of nationally representative data, although more research is needed to confirm these associations.

## 5. Conclusions

The prevalence of the double burden of malnutrition at the population level among schooled adolescents living in South Asia is a nutritional problem of public health importance. This study highlights the urgent need for addressing the double burden of malnutrition in South Asian schooled adolescents and investigating further malnutrition risk and protective factors. Determining the magnitude of the double burden among adolescents is important for the development of appropriate double-duty actions to accelerate progress towards ending malnutrition in all its forms.

## Figures and Tables

**Table 1:** Descriptive characteristics of South Asian schooled adolescents aged 12–15 years of the Global School-based student Health Survey.

Country	Survey Year	Response Rate (%) ^1^	Sample Size	Girls (%)
Pakistan	2009	76	4998	25.1
Afghanistan	2014	79	1493	63.2
Bangladesh	2014	91	2753	61.6
India	2007	83	7327	45.3
Maldives	2009	80	1977	56.2
Nepal	2015	70	4615	54.9
Sri Lanka	2016	89	2228	56.6
Bhutan	2016	95	3287	58.1

^1^ Response rate from countries’ fact sheet [38].

**Table 2:** Prevalence * of stunting among South Asian schooled adolescents aged 12–15 years old.

Country	Stunting (Height-for-Age < 2 SDs)			
	All Students (%)	Boys (%)	Girls (%)	χ^2^ (*p*-Value) ^1^
Pakistan	3.90	2.32	6.34	49.43 (0.000)
Afghanistan	28.15	35.97	19.82	38.44 (0.071)
Bangladesh	13.52	12.53	15.14	3.42 (0.460)
India	9.65	8.60	11.07	10.45 (0.198)
Maldives	13.33	12.13	14.39	1.26 (0.543)
Nepal	24.09	24.45	23.77	0.25 (0.592)
Sri Lanka	24.99	25.49	24.57	0.11 (0.843)
Bhutan	7.58	7.90	7.32	0.37 (0.662)
South Asia	12.97	11.64	14.80	51.60 (0.059)

^1^ χ^2^ represents test between sex and stunting, * following WHO growth reference.

**Table 3:** Prevalence * of thinness among South Asian schooled adolescents aged 12–15 years.

Country	Thinness (BMI-for-Age < 2 SDs)			
	All Students (%)	Boys (%)	Girls (%)	χ^2^ (*p*-Value) ^1^
Pakistan	9.09	9.51	8.43	1.59 (0.575)
Afghanistan	2.51	2.34	2.69	0.15 (0.781)
Bangladesh	11.43	12.83	9.13	7.89 (0.262)
India	12.82	13.48	11.92	3.26 (0.263)
Maldives	17.86	16.73	18.86	0.89 (0.521)
Nepal	8.25	9.78	6.93	10.84 (0.029)
Sri Lanka	18.56	20.14	17.22	1.41 (0.369)
Bhutan	1.49	2.02	1.05	5.07 (0.001)
South Asia	10.78	11.85	9.29	39.80 (0.109)

^1^ χ^2^ represents test between sex and thinness, * following WHO growth reference.

**Table 4:** Prevalence * of overweight among South Asian schooled adolescents aged 12–15 years.

Country	Overweight (BMI-for-Age > 1 SDs)			
	All Students (%)	Boys (%)	Girls (%)	χ^2^ (*p*-Value) ^1^
Pakistan	7.98	6.48	10.31	22.96 (0.001)
Afghanistan	19.04	21.25	16.69	4.01 (0.333)
Bangladesh	11.3	12.71	8.99	8.03 (0.292)
India	12.96	14.36	11.05	14.57 (0.010)
Maldives	13.77	18.33	9.74	17.80 (0.020)
Nepal	9.53	10.81	8.43	6.63 (0.151)
Sri Lanka	13.22	13.94	12.6	0.39 (0.580)
Bhutan	16.34	12.89	19.16	22.84 (0.002)
South Asia	10.77	11.43	9.87	14.84 (0.352)

^1^ χ^2^ represents test between sex and overweight, * following WHO growth reference.

**Table 5:** Stratified prevalence of health behaviours among South Asian schooled adolescents aged 12–15 years, GSHS datasets.

	Pakistan (%)	Afghanistan (%)	Bangladesh (%)	India (%)	Maldives (%)	Nepal (%)	Sri Lanka (%)	Bhutan (%)	South Asia (%)	χ^2^ (*p*-Value) ^1^
**5 fruits/vegs per day**	9.84	15.35	16.32	14.81	11.39	9.01	23.67	23.53	14.33	401.75 (0.000)
**Loneliness**	11.87	28.83	10.90	8.23	13.87	5.89	7.49	11.38	10.07	337.99 (0.000)
**Anxiety**	8.21	21.84	4.47	7.61	13.22	3.83	3.87	6.88	5.91	413.56 (0.000)
**Tobacco use**	6.30	6.52	6.90	1.22	8.99	4.90	2.49	19.29	5.33	273.97 (0.004)
**Physically activity**	11.57	9.64	41.24	30.07	21.61	14.42	17.36	15.54	27.14	2385.96 (0.000)
**Active transportation**	61.48	70.16	67.86	56.70	52.41	57.94	60.79	45.94	62.93	259.43 (0.004)
**Sedentary behaviour**	8.19	23.34	14.93	22.84	42.45	9.78	35.08	28.06	15.83	1199.93 (0.000)
**Tooth brushing**	30.18	41.08	64.09	55.58	77.06	49.61	71.19	42.96	53.97	2151.41 (0.000)
**Washing hands before meals**	96.55	93.97	96.90	94.08	91.46	96.00	97.33	96.19	96.25	88.10 (0.066)
**Washing hands after toilet**	96.59	94.38	98.06	96.68	95.32	95.59	97.08	95.71	97.05	90.24 (0.213)
**Washing hands with soap**	91.97	88.37	95.04	87.02	92.76	95.15	92.94	94.71	92.93	359.35 (0.001)
**Friendships**	91.89	86.25	91.20	89.83	90.48	95.57	94.75	90.46	91.94	150.86 (0.000)
**Peer support**	39.36	63.09	55.41	41.53	51.41	53.46	49.89	39.98	49.38	562.33 (0.000)
**Parental involvement in school**	52.36	45.55	53.70	47.66	31.60	50.16	68.45	30.50	52.98	308.76 (0.000)
**Parental understanding**	54.64	51.97	47.67	62.39	35.16	53.75	63.17	42.44	53.44	377.37 (0.000)
**Parental bonding**	50.47	54.72	43.54	57.38	48.77	50.47	70.29	39.46	50.33	647.34 (0.000)

^1^ χ^2^ represents test between country and each health behaviour.

**Table 6:** Multivariable logistic regression model for adolescent stunting in South Asia, GSHS datasets.

	Stunting FULL MODEL						Stunting REDUCED MODEL					
	OR	Bootstrap Std.Err.	z	*p*	95% Conf. Interval		OR	Bootstrap Std.Err.	z	*p*	95% Conf. Interval	
**Age**												
12 years	1.00						1.00					
13 years	1.93	0.35	3.62	0.000	1.35	2.75	1.96	0.36	3.71	0.000	1.37	2.80
14 years	2.71	0.61	4.45	0.000	1.75	4.20	2.66	0.59	4.41	0.000	1.72	4.11
15 years	3.33	0.70	5.74	0.000	2.21	5.03	3.27	0.66	5.83	0.000	2.20	4.87
**Sex**												
Boy	1.00						1.00					
Girl	1.15	0.20	0.81	0.416	0.82	1.60	1.19	0.18	1.14	0.254	0.88	1.61
**Country**												
Pakistan	1.00						1.00					
Afghanistan	11.80	3.89	7.48	0.000	6.18	22.53	10.67	3.14	8.05	0.000	6.00	19.00
Bangladesh	4.85	1.31	5.86	0.000	2.86	8.23	4.29	1.09	5.73	0.000	2.61	7.07
India	2.97	0.67	4.84	0.000	1.91	4.61	2.73	0.60	4.60	0.000	1.78	4.19
Maldives	3.62	1.13	4.11	0.000	1.96	6.68	3.24	0.89	4.31	0.000	1.90	5.54
Nepal	9.01	2.05	9.66	0.000	5.77	14.08	8.99	2.02	9.77	0.000	5.79	13.97
Sri Lanka	8.96	2.00	9.85	0.000	5.79	13.87	8.30	1.81	9.68	0.000	5.41	12.73
Bhutan	2.39	0.52	4.04	0.000	1.57	3.65	2.21	0.45	3.88	0.000	1.48	3.29
**5 fruits and vegs**												
<5 per day	1.00											
5 or more per day	0.90	0.16	−0.60	0.548	0.63	1.27						
**Loneliness**												
Never/rarely/sometimes	1.00											
Often/always	0.85	0.17	−0.81	0.417	0.58	1.26						
**Anxiety**												
Never/rarely/sometimes	1.00											
Often/always	0.93	0.18	−0.38	0.704	0.63	1.36						
**Tobacco use**												
0 days	1.00						1.00					
1 or more days	0.54	0.22	−1.51	0.132	0.24	1.21	0.52	0.18	−1.90	0.058	0.26	1.02
**Physically activity**												
<7 days per week	1.00											
7 days per week	0.85	0.12	−1.13	0.257	0.64	1.13						
**Active transportation**												
<3 days per week	1.00											
3 or more days per week	1.00	0.14	−0.03	0.978	0.76	1.30						
**Sedentary behaviour**												
<3 h per day	1.00											
3 or more hours per day	0.78	0.14	−1.35	0.176	0.55	1.12						
**Tooth brushing**												
<2 times per day	1.00											
2 times or more per day	0.90	0.07	−1.28	0.201	0.77	1.06						
**Washing hands before meals**												
Never/rarely	1.00						1.00					
Sometimes/often/always	1.46	0.29	1.89	0.059	0.99	2.17	1.33	0.24	1.57	0.118	0.93	1.89
**Washing hands after toilet**												
Never/rarely	1.00											
Sometimes/often/always	1.10	0.27	0.37	0.708	0.67	1.79						
**Washing hands with soap**												
Never/rarely	1.00											
Sometimes/often/always	1.05	0.18	0.30	0.767	0.76	1.46						
**Friendships**												
no friends	1.00											
1 or more friend	0.85	0.15	−0.92	0.358	0.59	1.21						
**Peer support**												
Never/rarely/sometimes	1.00											
Often/always	0.76	0.07	−3.15	0.002	0.64	0.90	0.75	0.06	−3.45	0.001	0.64	0.88
**Parental involvement in school**												
Never/rarely/sometimes	1.00											
Often/always	0.95	0.10	−0.45	0.656	0.78	1.17						
**Parental understanding**												
Never/rarely/sometimes	1.00											
Often/always	1.09	0.12	0.80	0.426	0.88	1.34						
**Parental bonding**												
Never/rarely/sometimes	1.00											
Often/always	1.03	0.15	0.22	0.825	0.78	1.36						

OR = Odds ratio.

**Table 7:** Multivariable logistic regression model for adolescent thinness in South Asia, GSHS datasets.

	Thinness FULL MODEL						Thinness REDUCED MODEL					
	OR	Bootstrap Std.Err.	z	*p*	95% Conf. Interval		OR	Bootstrap Std.Err.	z	*p*	95% Conf. Interval	
**Age**												
12 years	1.00						1.00					
13 years	1.86	0.33	3.49	0.000	1.31	2.64	1.65	0.27	3.02	0.003	1.19	2.28
14 years	1.36	0.25	1.69	0.090	0.95	1.94	1.25	0.22	1.23	0.218	0.88	1.77
15 years	1.26	0.26	1.13	0.259	0.84	1.88	1.16	0.21	0.78	0.433	0.81	1.66
**Sex**												
Boy	1.00						1.00					
Girl	0.79	0.13	−1.46	0.143	0.58	1.08	0.76	0.11	−1.81	0.070	0.57	1.02
**Country**												
Pakistan	1.00						1.00					
Afghanistan	0.27	0.09	−4.07	0.000	0.14	0.51	0.29	0.08	−4.54	0.000	0.17	0.50
Bangladesh	1.57	0.51	1.39	0.165	0.83	2.97	1.48	0.46	1.25	0.211	0.80	2.73
India	1.93	0.34	3.72	0.000	1.36	2.73	1.71	0.28	3.24	0.001	1.24	2.37
Maldives	3.21	0.72	5.17	0.000	2.06	4.99	3.09	0.63	5.53	0.000	2.07	4.62
Nepal	1.10	0.20	0.49	0.622	0.76	1.58	0.99	0.17	−0.06	0.956	0.71	1.39
Sri Lanka	3.25	0.68	5.65	0.000	2.16	4.88	2.99	0.60	5.50	0.000	2.02	4.42
Bhutan	0.18	0.05	−6.38	0.000	0.11	0.30	0.18	0.05	−6.66	0.000	0.11	0.30
**5 fruits and vegs**												
<5 per day	1.00											
5 or more per day	0.86	0.13	−1.02	0.309	0.65	1.15						
**Loneliness**												
Never/rarely/sometimes	1.00											
Often/always	1.19	0.21	0.97	0.332	0.84	1.68						
**Anxiety**												
Never/rarely/sometimes	1.00											
Often/always	0.99	0.16	−0.07	0.942	0.71	1.37						
**Tobacco use**												
0 days	1.00											
1 or more days	1.37	0.34	1.27	0.203	0.84	2.21						
**Physically activity**												
<7 days per week	1.00											
7 days per week	0.92	0.13	−0.57	0.567	0.71	1.21						
**Active transportation**												
<3 days per week	1.00											
3 or more days per week	1.15	0.10	1.59	0.111	0.97	1.35						
**Sedentary behaviour**												
<3 h per day	1.00						1.00					
3 or more hours per day	0.63	0.10	−2.92	0.004	0.46	0.86	0.62	0.09	−3.28	0.001	0.46	0.82
**Tooth brushing**												
<2 times per day	1.00						1.00					
2 times or more per day	0.70	0.12	−2.16	0.031	0.50	0.97	0.72	0.09	−2.61	0.009	0.56	0.92
**Washing hands before meals**												
Never/rarely	1.00											
Sometimes/often/always	1.02	0.27	0.07	0.945	0.61	1.70						
**Washing hands after toilet**												
Never/rarely	1.00											
Sometimes/often/always	1.07	0.29	0.25	0.802	0.63	1.81						
**Washing hands with soap**												
Never/rarely	1.00											
Sometimes/often/always	1.14	0.30	0.52	0.606	0.69	1.90						
**Friendships**												
no friends	1.00											
1 or more friend	0.98	0.20	−0.10	0.918	0.66	1.45						
**Peer support**												
Never/rarely/sometimes	1.00											
Often/always	0.94	0.07	−0.73	0.467	0.81	1.10						
**Parental involvement in school**												
Never/rarely/sometimes	1.00											
Often/always	0.95	0.11	−0.41	0.684	0.76	1.19						
**Parental understanding**												
Never/rarely/sometimes	1.00											
Often/always	1.09	0.11	0.92	0.356	0.90	1.32						
**Parental bonding**												
Never/rarely/sometimes	1.00											
Often/always	0.89	0.08	−1.28	0.202	0.74	1.07						

OR = Odds ratio.

**Table 8:** Multivariable logistic regression model for adolescent overweight in South Asia, GSHS datasets.

	Overweight FULL MODEL						Overweight REDUCED MODEL					
	OR	Bootstrap Std.Err.	z	*p*	95% Conf. Interval		OR	Bootstrap Std.Err.	z	*p*	95% Conf. Interval	
**Age**												
12 years	1.00											
13 years	0.58	0.10	−3.25	0.001	0.42	0.81	0.61	0.11	−2.75	0.006	0.42	0.87
14 years	0.53	0.09	−3.58	0.000	0.38	0.75	0.53	0.10	−3.48	0.001	0.37	0.76
15 years	0.47	0.10	−3.49	0.000	0.30	0.71	0.48	0.10	−3.37	0.001	0.31	0.73
**Sex**												
Boy	1.00											
Girl	0.72	0.14	−1.69	0.091	0.49	1.05	0.74	0.13	−1.67	0.094	0.52	1.05
**Country**												
Pakistan	1.00											
Afghanistan	2.63	0.57	4.47	0.000	1.72	4.03	2.64	0.51	5.02	0.000	1.81	3.86
Bangladesh	1.27	0.36	0.84	0.401	0.73	2.22	1.31	0.38	0.92	0.356	0.74	2.32
India	1.45	0.26	2.06	0.039	1.02	2.06	1.46	0.24	2.33	0.020	1.06	2.02
Maldives	1.45	0.35	1.54	0.123	0.90	2.32	1.65	0.37	2.23	0.026	1.06	2.57
Nepal	0.96	0.19	−0.21	0.835	0.65	1.41	0.97	0.19	−0.14	0.886	0.66	1.42
Sri Lanka	1.53	0.33	1.95	0.051	1.00	2.33	1.59	0.35	2.11	0.035	1.03	2.44
Bhutan	2.23	0.37	4.77	0.000	1.60	3.10	2.23	0.34	5.25	0.000	1.65	3.01
**5 fruits and vegs**												
<5 per day	1.00											
5 or more per day	0.84	0.17	−0.87	0.384	0.57	1.24						
**Loneliness**												
Never/rarely/sometimes	1.00											
Often/always	1.07	0.19	0.40	0.688	0.76	1.52						
**Anxiety**												
Never/rarely/sometimes	1.00											
Often/always	0.63	0.09	−3.07	0.002	0.47	0.85						
**Tobacco use**												
0 days	1.00						1.00					
1 or more days	0.45	0.12	−3.00	0.003	0.27	0.76	0.44	0.11	−3.34	0.001	0.27	0.71
**Physically activity**												
<7 days per week	1.00											
7 days per week	0.93	0.19	−0.34	0.734	0.63	1.38						
**Active transportation**												
<3 days per week	1.00											
3 or more days per week	1.01	0.14	0.09	0.925	0.77	1.34						
**Sedentary behaviour**												
<3 h per day	1.00											
3 or more hours per day	1.29	0.21	1.56	0.118	0.94	1.78						
**Tooth brushing**												
<2 times per day	1.00						1.00					
2 times or more per day	1.50	0.21	2.99	0.003	1.15	1.97	1.43	0.17	2.96	0.003	1.13	1.82
**Washing hands before meals**												
Never/rarely	1.00											
Sometimes/often/always	1.11	0.26	0.46	0.648	0.71	1.74						
**Washing hands after toilet**												
Never/rarely/sometimes	1.00											
Often/always	1.13	0.28	0.51	0.612	0.70	1.83						
**Washing hands with soap**												
Never/rarely	1.00						1.00					
Sometimes/often/always	1.59	0.32	2.30	0.021	1.07	2.37	1.58	0.31	2.31	0.021	1.07	2.33
**Friendships**												
no friends	1.00											
1 or more friend	0.85	0.16	−0.89	0.375	0.59	1.22						
**Peer support**												
Never/rarely/sometimes	1.00											
Often/always	1.00	0.12	0.01	0.996	0.79	1.26						
**Parental involvement in school**												
Never/rarely/sometimes	1.00						1.00					
Often/always	0.80	0.10	−1.74	0.081	0.63	1.03	0.80	0.08	−2.21	0.027	0.66	0.98
**Parental understanding**												
Never/rarely/sometimes	1.00											
Often/always	1.00	0.15	0.02	0.982	0.75	1.34						
**Parental bonding**												
Never/rarely/sometimes	1.00											
Often/always	0.99	0.09	−0.14	0.891	0.83	1.17						

OR = Odds ratio.

## Data Availability

Datasets are available at https://www.who.int/teams/noncommunicable-diseases/surveillance/systems-tools/global-school-based-student-health-survey (accessed on 14 July 2021).

## Supplementary Materials

The following are available online at https://www.mdpi.com/article/10.3390/nu13082867/s1; Table S1: Survey questions from the Global School-based student Health Survey (GSHS) datasets used in this study, Table S2: Bivariate relationships between stunting and health behaviours among adolescents aged 12–15 years old in South Asia, GSHS datasets; Table S3: Bivariate relationships between thinness and health behaviours among adolescents aged 12–15 years old in South Asia, GSHS datasets; Table S4: Bivariate relationships between overweight and health behaviours among adolescents aged 12–15 years old in South Asia, GSHS datasets. Tables S5–S7: Multivariable logistic regression model for adolescent stunting/thinness/overweight in Pakistan, GSHS Pakistan 2009 dataset; Tables S8–S10: Multivariable logistic regression model for adolescent stunting/thinness/overweight in Afghanistan, GSHS Afghanistan 2014 dataset; Tables S11–S13: Multivariable logistic regression model for adolescent stunting/thinness/overweight in Bangladesh, GSHS Bangladesh 2014 dataset; Tables S14–S16: Multivariable logistic regression model for adolescent stunting/thinness/overweight in India, GSHS India 2007 dataset; Tables S17–S19: Multivariable logistic regression model for adolescent stunting/thinness/overweight in Maldives, GSHS Maldives 2009 dataset; Tables S20–S22: Multivariable logistic regression model for adolescent stunting/thinness/overweight in Nepal, GSHS Nepal 2015 dataset; Tables S23–S25: Multivariable logistic regression model for adolescent stunting/thinness/overweight in Sri Lanka, GSHS Sri Lanka 2016 dataset; Tables S26–S28: Multivariable logistic regression model for adolescent stunting/thinness/overweight in Bhutan, GSHS Bhutan 2016 dataset.
