# Supplementary material for: The Double Burden of Malnutrition and Associated Factors among South Asian Adolescents: Findings from the Global School-Based Student Health Survey
Source: Nutrients. 2021 Aug 20;13(8):2867. doi: 10.3390/nu13082867 (PMC8399808; doi:10.3390/nu13082867)
Supplement: Supplementary file 1 [file nutrients-13-02867-s001.zip › nutrients-1323460-supplementary.pdf]

Table S1: Survey questions from the Global School-based student Health Survey (GSHS) datasets used in this study.

Survey question	Coding[1]	Variable name
During the past 30 days, how many times per day did you usually eat fruit? During the past 30 days, how many times per day did you usually eat vegetables?	<5 fruits and vegs per day (0)<br>5 fruits and vegs or more per day (1)	5 fruits and vegs
During the past 12 months, how often have you felt lonely?	Never/rarely/sometimes (0)<br>Often/always (1)	Loneliness
During the past 12 months, how often have you been so worried about something that you could not sleep at night?	Never/rarely/sometimes (0)<br>Often/always (1)	Anxiety
During the past 30 days, on how many days did you smoke cigarettes?	0 days (0)<br>1 or more days (1)	Tobacco
During the past 7 days, on how many days were you physically active for a total of at least 60 minutes per day?	<7 days per week (0)<br>7 days per week (1)	Physical activity
During the past 7 days, on how many days did you walk or ride a bicycle to or from school?	<3 days per week (0)<br>3 or more days per week (1)	Active transportation
How much time do you spend during a typical or usual day sitting and watching television, playing computer games, talking with friends, or doing other sitting activities?	<3 hours per day (0)<br>3 or more hours per day (1)	Sedentary behaviour
During the past 30 days, how many times per day did you usually clean or brush your teeth?	<2 times per day (0)<br>2 times or more per day (1)	Tooth brushing
During the past 30 days, how often did you wash your hands before eating?	Never/rarely (0)<br>Sometimes/often/always (1)	Washing hands before meals
During the past 30 days, how often did you wash your hands after using the toilet or latrine?	Never/rarely (0)<br>Sometimes/often/always (1)	Washing hands after toilet
During the past 30 days, how often did you use soap when washing your hands?	Never/rarely (0)<br>Sometimes/often/always (1)	Washing hands with soap
How many close friends do you have?	No friends (0)<br>1 or more friend (1)	Friendships
During the past 30 days, how often were most of the students in your school kind and helpful?	Never/rarely/sometimes (0)<br>Often/always (1)	Peer support
During the past 30 days, how often did your parents or guardians check to see if your homework was done?	Never/rarely/sometimes (0)<br>Often/always (1)	Parental involvement in school
During the past 30 days, how often did your parents or guardians understand your problems and worries?	Never/rarely/sometimes (0)<br>Often/always (1)	Parental understanding
During the past 30 days, how often did your parents or guardians really know what you were doing with your free time?	Never/rarely/sometimes (0)<br>Often/always (1)	Parental bonding

[1] The cut-offs applied here were used in previous GSHS publications

Table S2: Bivariate relationships between stunting and health behaviours among adolescents aged 12-15 years old in South Asia, GSHS datasets.

	Stunting (height-for-age <2 SD)		
	No stunted, % (n^1/N^1)	Stunted, % (n^2/N^2)	p-value

**5 fruits and vegs**			
5 or more per day	16.15% (3,359/20,798)	14.03% (409/2,915)	0.391
**Loneliness**			
Often/always	10.43% (2,173/20,825)	10.56% (307/2,908)	0.173
**Anxiety**			
Often/always	7.35% (1,542/20,972)	7.56% (222/2,938)	0.034
**Tobacco**			
1 or more days	6.50 % (1,352/20,799)	4.18% (122/2,920)	0.039
**Physically activity**			
7 days per week	22.65% (4,692/20,713)	17.74% (516/2,909)	0.066
**Active transportation**			
3 or more days per week	48.76% (10,071/20,656)	46.64% (1,353/2,901)	0.750
**Sedentary behaviour**			
3 or more hours per day	18.85% (3,903/20,705)	17.98% (522/2,904)	0.489
**Tooth brushing**			
2 times or more per day	49.27% (10,329/20,963)	51.35% (1,507/2,935)	0.282
**Washing hands before meals**			
Sometimes/often/always	95.70% (19,978/20,875)	95.48% (2,787/2,919)	0.065
**Washing hands after toilet**			
Sometimes/often/always	96.45% (20,124/20,865)	96.27% (2,815/2,924)	0.469
**Washing hands with soap**			
Sometimes/often/always	91.72% (19,164/20,893)	92.39% (2,706/2,929)	0.052
**Friendships**			
1 or more friend	91.70% (19,107/20,836)	91.28% (2,659/2,913)	0.483
**Peer support**			
Often/always	47.39% (9,723/20,515)	48.23% (1,392/2,886)	0.226
**Parental involvement**			
Often/always	48.38% (9,974/20,614)	49.20% (1,417/2,880)	0.275
**Parental understanding**			
Often/always	54.13% (11,090/20,487)	54.24% (1,553/2,863)	0.906
**Parental bonding**			
Often/always	51.97% (10,564/20,328)	52.76% (1,499/2,841)	0.646

n^1 refers to the number of South Asian adolescents based on GSHS datasets who are no stunted and fall into the health behaviour

N^1 refers to the number of South Asian adolescents based on GSHS datasets who are no stunted

n^2 refers to the number of South Asian adolescents based on GSHS datasets who are stunted and fall into the health behaviour

N^2 refers to the number of South Asian adolescents based on GSHS datasets who are stunted

Table S3: Bivariate relationships between thinness and health behaviours among adolescents aged 12-15 years old in South Asia, GSHS datasets.

	**Thinness (BMI-for-age <2 SD)**		
	**No thin, % (n/N)**	**Thin, % (n/N)**	**p-value**
**5 fruits and vegs**			
5 or more per day	16.15% (3,467/21,466)	13.40% (301/2,247)	0.361
**Loneliness**			
Often/always	10.58% (2,272/21,478)	9.22% (208/2,255)	0.532
**Anxiety**			
Often/always	7.52% (1,628/21,644)	6.00% (136/2,266)	0.261
**Tobacco**			
1 or more days	6.37% (1,367/21,467)	4.75% (107/2,252)	0.457
**Physically activity**			
7 days per week	22.00% (4,706/21,387)	22.46% (502/2,235)	0.837
**Active transportation**			
3 or more days per week	48.24% (10,290/21,329)	50.90% (1,134/2,228)	0.311
**Sedentary behaviour**			
3 or more hours per day	18.90% (4,041/21,380)	17.23% (384/2,229)	0.012
**Tooth brushing**			
2 times or more per day	49.38% (10,679/21,627)	50.95% (1,157/2,271)	0.056
**Washing hands before meals**			
Sometimes/often/always	95.76% (20,622/21,534)	94.84% (2,146/2,260)	0.971
**Washing hands after toilet**			
Sometimes/often/always	96.38% (20,756/21,536)	96.89% (2,183/2,253)	0.553
**Washing hands with soap**			
Sometimes/often/always	91.91% (19,814/21,557)	90.77% (2,056/2,265)	0.984
**Friendships**			
1 or more friend	91.71% (19,716/21,498)	91,07% (2,050/2,251)	0.683
**Peer support**			
Often/always	47.81 (10,129/21,186)	44.51% (986/2,215)	0.304
**Parental involvement**			
Often/always	48.32% (10,277/21,269)	50.07% (1,114/2,225)	0.946
**Parental understanding**			
Often/always	54,01% (11,414/21,132)	55.41% (1,229/2,218)	0.990
**Parental bonding**			
Often/always	51.88% (10,888/20,985)	53.88% (1,175/2,184)	0.253

n[1] refers to the number of South Asian adolescents based on GSHS datasets who are no stunted and fall into the health behaviour

N[1] refers to the number of South Asian adolescents based on GSHS datasets who are no stunted

n[2] refers to the number of South Asian adolescents based on GSHS datasets who are stunted and fall into the health behaviour

N[2] refers to the number of South Asian adolescents based on GSHS datasets who are stunted

Table S4: Bivariate relationships between overweight and health behaviours among adolescents aged 12-15 years old in South Asia, GSHS datasets.

	**Overweight (BMI-for-age >1 SD)**		
	**No overweight, % (n/N)**	**Overweight, % (n/N)**	**p-value**
**5 fruits and vegs**			
5 or more per day	15.69% (3,313/21,118)	17.53% (455/2,595)	0.417
**Loneliness**			
Often/always	10.25% (2,167/21,142)	12.08% (313/2,591)	0.647
**Anxiety**			
Often/always	7.23% (1,539/21,293)	8.60% (225/2,617)	0.151
**Tobacco**			
1 or more days	6.26% (1,323/21,121)	5.81% (151/2,598)	0.001
**Physically activity**			
7 days per week	22.26% (4,684/21,046)	20.34% (524/2,576)	0.981
**Active transportation**			
3 or more days per week	48.84% (10,254/20,993)	45.63% (1,170/2,564)	0.972
**Sedentary behaviour**			
3 or more hours per day	18.17% (3,822/21,034)	23.42% (603/2,575)	0.009
**Tooth brushing**			
2 times or more per day	49.10% (10,450/21,284)	52.98% (1,385/2,614)	0.005
**Washing hands before meals**			
Sometimes/often/always	95.69% (20,274/21,187)	95.55% (2,491/2,607)	0.392
**Washing hands after toilet**			
Sometimes/often/always	96.45% (20,427/21,178)	96.21% (2,512/2,611)	0.091
**Washing hands with soap**			
Sometimes/often/always	91.71% (19,454/21,213)	92.60% (2,416/2,609)	0.102
**Friendships**			
1 or more friend	91% (19,406/21,148)	90.73% (2,360/2,601)	0.141
**Peer support**			
Often/always	47.78% (9,963/20,850)	45.16% (1,152/2,551)	0.610
**Parental involvement**			

Often/always	48.94% (10,247/20,939)	44.77% (1,144/2,555)	0.061
**Parental understanding**			
Often/always	54.40% (11,321/20,810)	52.05% (2,540/1,322)	0.832
**Parental bonding**			
Often/always	52.11% (10,764/20,655)	51.67% (1,299/2,514)	0.837

n^1 refers to the number of South Asian adolescents based on GSHS datasets who are no stunted and fall into the health behaviour

N^1 refers to the number of South Asian adolescents based on GSHS datasets who are no stunted

n^2 refers to the number of South Asian adolescents based on GSHS datasets who are stunted and fall into the health behaviour

N^2 refers to the number of South Asian adolescents based on GSHS datasets who are stunted

Table S5: Multivariable logistic regression model for adolescent stunting in Pakistan, GSHS Pakistan 2009 dataset.

	Stunting (height-for-age <2 SD) FULL MODEL						Stunting (height-for-age <2 SD) REDUCED MODEL					
	**OR**	**Bootstrap std.err.**	**z**	**p**	**95% conf. interval**		**OR**	**Bootstrap std.err.**	**z**	**p**	**95% conf. interval**	
**Age**												
12- 13 years	1.00						1.00					
14 - 15 years	1.16	0.13	1.26	0.208	0.92	1.46	1.16	0.14	1.25	0.211	0.92	1.47
**Sex**												
Boy	1.00						1.00					
Girl	3.08	0.75	4.60	0.000	1.91	4.98	2.79	0.72	3.99	0.000	1.69	4.63
**5 fruits and vegs**												
<5 per day	1.00											
5 or more per day	1.19	0.32	0.66	0.512	0.71	2.00						
**Loneliness**												
Never/rarely/sometimes	1.00											
Often/always	1.00	0.20	0.00	0.998	0.67	1.48						
**Anxiety**												
Never/rarely/sometimes	1.00											
Often/always	1.30	0.48	0.70	0.484	0.63	2.69						
**Tobacco**												
0 days	1.00											

1 or more days	0.57	0.26	-1.24	0.214	0.23	1.39						
**Physically activity**												
<7 days per week	1.00											
7 days per week	0.66	0.22	-1.25	0.212	0.34	1.27						
**Active transportation**												
<3 days per week	1.00											
3 or more days per week	1.35	0.27	1.51	0.132	0.91	1.99						
**Sedentary behaviour**												
<3 hours per day	1.00											
3 or more hours per day	0.89	0.56	-0.19	0.849	0.25	3.09						
**Tooth brushing**												
<2 times per day	1.00											
2 times or more per day	0.97	0.16	-0.21	0.835	0.70	1.34						
**Washing hands before meals**												
Never/rarely	1.00											
Sometimes/often/always	0.84	0.58	-0.25	0.802	0.22	3.23						
**Washing hands after toilet**												
Never/rarely	1.00											
Sometimes/often/always	0.53	0.41	-0.83	0.408	0.12	2.37						
**Washing hands with soap**												
Never/rarely	1.00						1.00					
Sometimes/often/always	3.44	2.21	1.93	0.053	0.98	12.08	1.54	0.95	0.70	0.485	0.46	5.18

**Friendships**												
no friends	1.00											
1 or more friend	0.69	0.31	-0.84	0.404	0.28	1.66						
**Peer support**												
Never/rarely/sometimes	1.00											
Often/always	1.01	0.25	0.04	0.966	0.62	1.64						
**Parental involvement in school**												
Never/rarely/sometimes	1.00											
Often/always	0.89	0.12	-0.83	0.407	0.68	1.17						
**Parental understanding**												
Never/rarely/sometimes	1.00											
Often/always	0.94	0.25	-0.24	0.808	0.55	1.59						
**Parental bonding**												
Never/rarely/sometimes	1.00											
Often/always	0.91	0.30	-0.53	0.638	0.62	1.34						

Table S6: Multivariable logistic regression model for adolescent thinness in Pakistan, GSHS Pakistan 2009 dataset.

	**Thinness (BMI-for-age <2 SD) FULL MODEL**						**Thinness (BMI-for-age <2 SD) REDUCED MODEL**					
	**OR**	**Bootstrap std.err.**	**z**	**p**	**95% conf. interval**		**OR**	**Bootstrap std.err.**	**z**	**p**	**95% conf. interval**	
**Age**												

12- 13 years	1.00						1.00					
14 - 15 years	0.90	0.08	-1.11	0.267	0.75	1.08	0.92	0.09	-0.90	0.367	0.76	1.11
**Sex**												
Boy	1.00						1.00					
Girl	1.00	0.28	0.00	0.998	0.58	1.73	0.86	0.23	-0.57	0.571	0.51	1.45
**5 fruits and vegs**												
<5 per day	1.00											
5 or more per day	1.15	0.21	0.78	0.437	0.80	1.66						
**Loneliness**												
Never/rarely/sometimes	1.00											
Often/always	1.35	0.31	1.31	0.189	0.86	2.11						
**Anxiety**												
Never/rarely/sometimes	1.00						1.00					
Often/always	0.55	0.12	-2.69	0.007	0.36	0.85	0.64	0.15	-1.86	0.063	0.40	1.03
**Tobacco**												
0 days	1.00											
1 or more days	1.37	0.31	1.40	0.161	0.88	2.13						
**Physically activity**												
<7 days per week	1.00											
7 days per week	1.08	0.26	0.31	0.753	0.67	1.72						
**Active transportation**												
<3 days per week	1.00											

3 or more days per week	1.02	0.17	0.13	0.894	0.74	1.41						
**Sedentary behaviour**												
<3 hours per day	1.00						1.00					
3 or more hours per day	0.47	0.16	-2.18	0.029	0.24	0.93	0.56	0.20	-1.63	0.103	0.28	1.12
**Tooth brushing**												
<2 times per day	1.00											
2 times or more per day	0.78	0.12	-1.67	0.096	0.58	1.05						
**Washing hands before meals**												
Never/rarely	1.00											
Sometimes/often/always	0.82	0.30	-0.54	0.591	0.40	1.69						
**Washing hands after toilet**												
Never/rarely	1.00											
Sometimes/often/always	1.22	0.52	0.47	0.639	0.53	2.83						
**Washing hands with soap**												
Never/rarely	1.00											
Sometimes/often/always	0.91	0.24	-0.35	0.726	0.54	1.54						
**Friendships**												
no friends	1.00											
1 or more friend	1.06	0.27	0.22	0.827	0.64	1.76						
**Peer support**												
Never/rarely/sometimes	1.00											
Often/always	1.20	0.16	1.35	0.178	0.92	1.56						

**Parental involvement in school**												
Never/rarely/sometimes	1.00											
Often/always	0.81	0.12	-1.45	0.147	0.62	1.08						
**Parental understanding**												
Never/rarely/sometimes	1.00											
Often/always	1.03	0.18	0.18	0.858	0.73	1.46						
**Parental bonding**												
Never/rarely/sometimes	1.00											
Often/always	1.15	0.14	1.17	0.243	0.91	1.46						

Table S7: Multivariable logistic regression model for adolescent overweight in Pakistan, GSHS Pakistan 2009 dataset.

	**Overweight (BMI-for-age >1 SD) FULL MODEL**						**Overweight (BMI-for-age >1 SD) REDUCED MODEL**					
	**OR**	**Bootstrap std.err.**	**z**	**p**	**95% conf. interval**		**OR**	**Bootstrap std.err.**	**z**	**p**	**95% conf. interval**	
**Age**												
12- 13 years	1.00						1.00					
14 - 15 years	0.99	0.08	-0.14	0.887	0.85	1.15	0.99	0.07	-0.11	0.909	0.87	1.13
**Sex**												
Boy	1.00						1.00					
Girl	1.55	0.31	2.23	0.026	1.06	2.29	1.64	0.30	2.70	0.007	1.15	2.35

Supplementary material

**5 fruits and vegs**							
<5 per day	1.00						
5 or more per day	0.95	0.20	-0.26	0.794	0.62	1.44	
**Loneliness**							
Never/rarely/sometimes	1.00						
Often/always	1.02	0.23	0.09	0.930	0.66	1.58	
**Anxiety**							
Never/rarely/sometimes	1.00						
Often/always	0.68	0.23	-1.14	0.254	0.36	1.31	
**Tobacco**							
0 days	1.00						
1 or more days	0.90	0.34	-0.28	0.776	0.42	1.90	
**Physically activity**							
<7 days per week	1.00						
7 days per week	0.73	0.21	-1.08	0.279	0.41	1.29	
**Active transportation**							
<3 days per week	1.00						
3 or more days per week	0.88	0.11	-1.03	0.305	0.70	1.12	
**Sedentary behaviour**							
<3 hours per day	1.00						
3 or more hours per day	1.27	0.25	1.21	0.225	0.86	1.86	
**Tooth brushing**							

<2 times per day	1.00						1.00					
2 times or more per day	1.50	0.35	1.73	0.084	0.95	2.37	1.48	0.28	2.06	0.040	1.02	2.14
**Washing hands before meals**												
Never/rarely	1.00											
Sometimes/often/always	0.72	0.33	-0.72	0.474	0.30	1.75						
**Washing hands after toilet**												
Never/rarely	1.00											
Sometimes/often/always	2.42	1.89	1.13	0.259	0.52	11.18						
**Washing hands with soap**												
Never/rarely	1.00											
Sometimes/often/always	1.39	0.58	0.79	0.432	0.61	3.16						
**Friendships**												
no friends	1.00											
1 or more friend	1.22	0.38	0.63	0.530	0.66	2.24						
**Peer support**												
Never/rarely/sometimes	1.00											
Often/always	0.82	0.15	-1.10	0.272	0.58	1.17						
**Parental involvement in school**												
Never/rarely/sometimes	1.00											
Often/always	1.13	0.17	0.81	0.418	0.84	1.52						
**Parental understanding**												
Never/rarely/sometimes	1.00						1.00					

Often/always	0.71	0.08	-2.98	0.003	0.56	0.89	0.71	0.08	-3.00	0.003	0.56	0.89
**Parental bonding**												
Never/rarely/sometimes	1.00											
Often/always	0.91	0.12	-0.77	0.443	0.70	1.17						

Table S8: Multivariable logistic regression model for adolescent stunting in Afghanistan, GSHS Afghanistan 2014 dataset.

	**Stunting (height-for-age <2 SD) FULL MODEL**						**Stunting (height-for-age <2 SD) REDUCED MODEL**					
	**OR**	**Bootstrap std.err.**	**z**	**p**	**95% conf. interval**		**OR**	**Bootstrap std.err.**	**z**	**p**	**95% conf. interval**	
**Age**												
12- 13 years	1.00						1.00					
14 - 15 years	0.93	0.11	-0.58	0.560	0.74	1.17	0.93	0.10	-0.62	0.535	0.75	1.16
**Sex**												
Boy	1.00						1.00					
Girl	0.32	0.14	-2.67	0.008	0.13	0.74	0.42	0.18	-1.98	0.048	0.18	0.99
**5 fruits and vegs**												
<5 per day	1.00											
5 or more per day	0.67	0.30	-0.90	0.369	0.29	1.59						
**Loneliness**												
Never/rarely/sometimes	1.00											

Supplementary material

Often/always	1.06	0.19	0.31	0.756	0.74	1.50						
**Anxiety**												
Never/rarely/sometimes	1.00											
Often/always	1.58	0.60	1.20	0.230	0.75	3.34						
**Tobacco**												
0 days	1.00											
1 or more days	0.36	0.17	-2.18	0.029	0.14	0.90						
**Physically activity**												
<7 days per week	1.00											
7 days per week	1.03	0.49	0.07	0.948	0.41	2.61						
**Active transportation**												
<3 days per week	1.00						1.00					
3 or more days per week	1.38	0.22	1.96	0.050	1.00	1.89	1.46	0.22	2.46	0.014	1.08	1.97
**Sedentary behaviour**												
<3 hours per day	1.00						1.00					
3 or more hours per day	2.03	0.69	2.06	0.039	1.04	3.97	2.00	0.50	2.78	0.005	1.23	3.26
**Tooth brushing**												
<2 times per day	1.00						1.00					
2 times or more per day	0.48	0.10	-3.53	0.000	0.32	0.72	0.43	0.09	-3.98	0.000	0.28	0.65
**Washing hands before meals**												
Never/rarely	1.00											
Sometimes/often/always	0.50	0.68	-0.51	0.611	0.04	7.10						

**Washing hands after toilet**							
Never/rarely	1.00						
Sometimes/often/always	0.53	0.35	-0.95	0.343	0.14	1.97	
**Washing hands with soap**							
Never/rarely	1.00						
Sometimes/often/always	1.12	0.62	0.20	0.840	0.38	3.29	
**Friendships**							
no friends	1.00						
1 or more friend	0.95	0.58	-0.09	0.927	0.29	3.13	
**Peer support**							
Never/rarely/sometimes	1.00						
Often/always	0.82	0.20	-0.83	0.409	0.51	1.32	
**Parental involvement in school**							
Never/rarely/sometimes	1.00						
Often/always	0.80	0.25	-0.71	0.476	0.43	1.48	
**Parental understanding**							
Never/rarely/sometimes	1.00						
Often/always	0.75	0.20	-1.09	0.277	0.45	1.26	
**Parental bonding**							
Never/rarely/sometimes	1.00						
Often/always	1.47	0.52	1.10	0.272	0.74	2.94	

Table S9: Multivariable logistic regression model for adolescent thinness in Afghanistan, GSHS Afghanistan 2014 dataset.

	Thinness (BMI-for-age <2 SD) FULL MODEL						Thinness (BMI-for-age <2 SD) REDUCED MODEL					
	**OR**	**Bootstrap std.err.**	**z**	**p**	**95% conf. interval**		**OR**	**Bootstrap std.err.**	**z**	**p**	**95% conf. interval**	
**Age**												
12- 13 years	1.00						1.00					
14 - 15 years	1.40	0.56	0.84	0.399	0.64	3.08	1.38	0.27	1.65	0.099	0.94	2.03
**Sex**												
Boy	1.00						1.00					
Girl	1.37	1.11	0.39	0.695	0.28	6.73	1.13	0.63	0.22	0.830	0.38	3.38
**5 fruits and vegs**												
<5 per day	1.00											
5 or more per day	0.93	0.81	-0.09	0.931	0.17	5.10						
**Loneliness**												
Never/rarely/sometimes	1.00											
Often/always	0.45	0.36	-1.00	0.318	0.10	2.15						
**Anxiety**												
Never/rarely/sometimes	1.00											
Often/always	1.25	1.05	0.27	0.788	0.24	6.47						
**Tobacco**												
0 days	1.00											

1 or more days	1.00	(omitted)										
**Physically activity**												
<7 days per week	1.00											
7 days per week	0.60	0.35	-0.87	0.382	0.19	1.87						
**Active transportation**												
<3 days per week	1.00											
3 or more days per week	1.33	0.64	0.59	0.554	0.52	3.43						
**Sedentary behaviour**												
<3 hours per day	1.00											
3 or more hours per day	1.66	1.52	0.56	0.578	0.28	10.00						
**Tooth brushing**												
<2 times per day	1.00						1.00					
2 times or more per day	2.53	1.51	1.55	0.120	0.79	8.16	2.43	0.69	3.11	0.002	1.39	4.25
**Washing hands before meals**												
Never/rarely	1.00											
Sometimes/often/always	1.00	(omitted)										
**Washing hands after toilet**												
Never/rarely	1.00											
Sometimes/often/always	1.00	(omitted)										
**Washing hands with soap**												
Never/rarely	1.00											
Sometimes/often/always	1.00	(omitted)										

**Friendships**												
no friends	1.00											
1 or more friend	1.00	(omitted)										
**Peer support**												
Never/rarely/sometimes	1.00						1.00					
Often/always	0.21	0.13	-2.45	0.014	0.06	0.73	0.38	0.14	-2.63	0.009	0.18	0.78
**Parental involvement in school**												
Never/rarely/sometimes	1.00											
Often/always	1.64	1.03	0.79	0.432	0.48	5.61						
**Parental understanding**												
Never/rarely/sometimes	1.00											
Often/always	1.75	1.28	0.76	0.446	0.42	7.31						
**Parental bonding**												
Never/rarely/sometimes	1.00											
Often/always	1.29	0.94	0.34	0.732	0.30	5.43						

Table S10: Multivariable logistic regression model for adolescent overweight in Afghanistan, GSHS Afghanistan 2014 dataset.

	**Overweight (BMI-for-age >1 SD) FULL MODEL**						**Overweight (BMI-for-age >1 SD) REDUCED MODEL**					
	**OR**	**Bootstrap std.err.**	**z**	**p**	**95% conf. interval**		**OR**	**Bootstrap std.err.**	**z**	**p**	**95% conf. interval**	

Supplementary material

**Age**												
12- 13 years	1.00						1.00					
14 - 15 years	0.78	0.14	-1.39	0.166	0.55	1.11	0.83	0.14	-1.08	0.282	0.59	1.17
**Sex**												
Boy	1.00						1.00					
Girl	0.56	0.19	-1.68	0.092	0.29	1.10	0.65	0.20	-1.41	0.159	0.36	1.18
**5 fruits and vegs**												
<5 per day	1.00											
5 or more per day	0.92	0.32	-0.25	0.805	0.46	1.83						
**Loneliness**												
Never/rarely/sometimes	1.00											
Often/always	0.84	0.16	-0.93	0.350	0.58	1.22						
**Anxiety**												
Never/rarely/sometimes	1.00						1.00					
Often/always	2.26	0.85	2.16	0.031	1.08	4.73	1.60	0.40	1.86	0.062	0.98	2.61
**Tobacco**												
0 days	1.00											
1 or more days	0.67	0.54	-0.5	0.618	0.14	3.28						
**Physically activity**												
<7 days per week	1.00											
7 days per week	1.44	0.47	1.13	0.260	0.76	2.72						
**Active transportation**												

<3 days per week	1.00											
3 or more days per week	0.86	0.14	-0.95	0.343	0.63	1.18						
**Sedentary behaviour**												
<3 hours per day	1.00											
3 or more hours per day	0.92	0.31	-0.26	0.796	0.47	1.78						
**Tooth brushing**												
<2 times per day	1.00											
2 times or more per day	0.82	0.18	-0.89	0.371	0.54	1.26						
**Washing hands before meals**												
Never/rarely	1.00						1.00					
Sometimes/often/always	0.23	0.17	-2.04	0.041	0.06	0.94	0.46	0.18	-1.95	0.051	0.21	1.00
**Washing hands after toilet**												
Never/rarely	1.00											
Sometimes/often/always	1.57	0.82	0.87	0.383	0.57	4.37						
**Washing hands with soap**												
Never/rarely	1.00											
Sometimes/often/always	2.29	1.42	1.34	0.181	0.68	7.73						
**Friendships**												
no friends	1.00						1.00					
1 or more friend	0.46	0.12	-2.88	0.004	0.28	0.78	0.71	0.18	-1.34	0.182	0.43	1.17
**Peer support**												
Never/rarely/sometimes	1.00											

Often/always	1.15	0.18	0.93	0.353	0.85	1.56						
**Parental involvement in school**												
Never/rarely/sometimes	1.00											
Often/always	1.16	0.28	0.63	0.526	0.73	1.85						
**Parental understanding**												
Never/rarely/sometimes	1.00											
Often/always	0.57	0.19	-1.68	0.093	0.30	1.10						
**Parental bonding**												
Never/rarely/sometimes	1.00											
Often/always	1.18	0.30	0.67	0.500	0.73	1.93						

Table S11: Multivariable logistic regression model for adolescent stunting in Bangladesh, GSHS Bangladesh 2014 dataset.

	**Stunting (height-for-age <2 SD) FULL MODEL**						**Stunting (height-for-age <2 SD) REDUCED MODEL**					
	**OR**	**Bootstrap std.err.**	**z**	**p**	**95% conf. interval**		**OR**	**Bootstrap std.err.**	**z**	**p**	**95% conf. interval**	
**Age**												
12- 13 years	1.00						1.00					
14 - 15 years	1.74	0.39	2.45	0.014	1.12	2.70	1.61	0.36	2.10	0.036	1.03	2.50
**Sex**												
Boy	1.00						1.00					
Girl	1.12	0.43	0.30	0.766	0.53	2.38	1.31	0.44	0.82	0.411	0.68	2.52

Supplementary material

**5 fruits and vegs**							
<5 per day	1.00						
5 or more per day	0.76	0.32	-0.66	0.509	0.33	1.72	
**Loneliness**							
Never/rarely/sometimes	1.00						
Often/always	0.68	0.30	-0.89	0.376	0.29	1.59	
**Anxiety**							
Never/rarely/sometimes	1.00						
Often/always	0.64	0.44	-0.65	0.514	0.17	2.42	
**Tobacco**							
0 days	1.00						
1 or more days	0.56	0.61	-0.53	0.594	0.06	4.84	
**Physically activity**							
<7 days per week	1.00						
7 days per week	0.90	0.24	-0.41	0.680	0.53	1.50	
**Active transportation**							
<3 days per week	1.00						
3 or more days per week	0.92	0.30	-0.25	0.804	0.48	1.76	
**Sedentary behaviour**							
<3 hours per day	1.00						
3 or more hours per day	0.70	0.31	-0.81	0.418	0.30	1.65	
**Tooth brushing**							

<2 times per day	1.00											
2 times or more per day	0.89	0.15	-0.70	0.485	0.65	1.23						
**Washing hands before meals**												
Never/rarely	1.00						1.00					
Sometimes/often/always	4.48	2.73	2.46	0.014	1.35	14.80	4.76	3.02	2.46	0.014	1.37	16.53
**Washing hands after toilet**												
Never/rarely	1.00											
Sometimes/often/always	1.54	1.16	0.57	0.568	0.35	6.74						
**Washing hands with soap**												
Never/rarely	1.00											
Sometimes/often/always	1.30	1.12	0.30	0.762	0.24	7.04						
**Friendships**												
no friends	1.00											
1 or more friend	0.87	0.32	-0.38	0.706	0.42	1.79						
**Peer support**												
Never/rarely/sometimes	1.00						1.00					
Often/always	0.56	0.10	-3.10	0.002	0.39	0.81	0.57	0.12	-2.71	0.007	0.38	0.86
**Parental involvement in school**												
Never/rarely/sometimes	1.00											
Often/always	0.88	0.18	-0.62	0.536	0.60	1.31						
**Parental understanding**												
Never/rarely/sometimes	1.00											

Often/always	1.25	0.24	1.14	0.255	0.85	1.82
**Parental bonding**						
Never/rarely/sometimes	1.00					
Often/always	1.28	0.43	0.74	0.462	0.67	2.46

Table S12: Multivariable logistic regression model for adolescent thinness in Bangladesh, GSHS Bangladesh 2014 dataset.

	**Thinness (BMI-for-age <2 SD) FULL MODEL**						**Thinness (BMI-for-age <2 SD) REDUCED MODEL**					
	**OR**	**Bootstrap std.err.**	**z**	**p**	**95% conf. interval**		**OR**	**Bootstrap std.err.**	**z**	**p**	**95% conf. interval**	
**Age**												
12- 13 years	1.00						1.00					
14 - 15 years	0.69	0.17	-1.56	0.120	0.43	1.10	0.71	0.14	-1.69	0.092	0.48	1.06
**Sex**												
Boy	1.00						1.00					
Girl	0.67	0.27	-1.00	0.317	0.31	1.46	0.65	0.23	-1.23	0.219	0.32	1.30
**5 fruits and vegs**												
<5 per day	1.00											
5 or more per day	0.67	0.22	-1.22	0.223	0.35	1.28						
**Loneliness**												
Never/rarely/sometimes	1.00											
Often/always	1.13	0.48	0.30	0.765	0.50	2.59						

Supplementary material

**Anxiety**												
Never/rarely/sometimes	1.00											
Often/always	1.84	0.71	1.58	0.115	0.86	3.93						
**Tobacco**												
0 days	1.00											
1 or more days	1.81	0.85	1.26	0.206	0.72	4.54						
**Physically activity**												
<7 days per week	1.00											
7 days per week	0.93	0.25	-0.26	0.797	0.55	1.59						
**Active transportation**												
<3 days per week	1.00											
3 or more days per week	1.25	0.26	1.10	0.272	0.84	1.88						
**Sedentary behaviour**												
<3 hours per day	1.00						1.00					
3 or more hours per day	0.46	0.19	-1.88	0.060	0.20	1.03	0.40	0.15	-2.51	0.012	0.20	0.82
**Tooth brushing**												
<2 times per day	1.00						1.00					
2 times or more per day	0.53	0.20	-1.72	0.085	0.25	1.09	0.57	0.14	-2.20	0.028	0.35	0.94
**Washing hands before meals**												
Never/rarely	1.00											
Sometimes/often/always	1.46	1.85	0.30	0.765	0.12	17.43						
**Washing hands after toilet**												

Never/rarely	1.00						
Sometimes/often/always	0.60	0.86	-0.36	0.721	0.04	9.97	
**Washing hands with soap**							
Never/rarely	1.00						
Sometimes/often/always	1.93	2.44	0.52	0.604	0.16	22.98	
**Friendships**							
no friends	1.00						
1 or more friend	1.15	0.51	0.31	0.754	0.48	2.72	
**Peer support**							
Never/rarely/sometimes	1.00						
Often/always	1.03	0.19	0.18	0.855	0.72	1.49	
**Parental involvement in school**							
Never/rarely/sometimes	1.00						
Often/always	1.00	0.25	0.00	0.997	0.62	1.62	
**Parental understanding**							
Never/rarely/sometimes	1.00						
Often/always	1.12	0.23	0.53	0.595	0.74	1.69	
**Parental bonding**							
Never/rarely/sometimes	1.00						
Often/always	0.75	0.14	-1.52	0.129	0.52	1.09	

Table S13: Multivariable logistic regression model for adolescent overweight in Bangladesh, GSHS Bangladesh 2014 dataset.

	Overweight (BMI-for-age >1 SD) FULL MODEL						Overweight (BMI-for-age >1 SD) REDUCED MODEL					
	OR	Bootstrap std.err.	z	p	95% conf. interval		OR	Bootstrap std.err.	z	p	95% conf. interval	
**Age**												
12- 13 years	1.00						1.00					
14 - 15 years	0.88	0.23	-0.49	0.626	0.52	1.48	0.92	0.22	-0.34	0.735	0.59	1.46
**Sex**												
Boy	1.00						1.00					
Girl	0.50	0.22	-1.57	0.118	0.21	1.19	0.56	0.21	-1.51	0.130	0.27	1.18
**5 fruits and vegs**												
<5 per day	1.00											
5 or more per day	0.71	0.43	-0.56	0.574	0.22	2.33						
**Loneliness**												
Never/rarely/sometimes	1.00											
Often/always	1.11	0.36	0.32	0.748	0.59	2.09						
**Anxiety**												
Never/rarely/sometimes	1.00											
Often/always	0.19	0.09	-3.45	0.001	0.07	0.48						
**Tobacco**												
0 days	1.00						1.00					

1 or more days	0.28	0.17	-2.13	0.033	0.09	0.91	0.20	0.09	-3.72	0.000	0.08	0.46
**Physically activity**												
<7 days per week	1.00											
7 days per week	0.89	0.34	-0.32	0.750	0.42	1.87						
**Active transportation**												
<3 days per week	1.00											
3 or more days per week	1.13	0.36	0.39	0.695	0.60	2.13						
**Sedentary behaviour**												
<3 hours per day	1.00											
3 or more hours per day	1.33	0.39	0.97	0.330	0.75	2.38						
**Tooth brushing**												
<2 times per day	1.00						1.00					
2 times or more per day	2.19	0.68	2.51	0.012	1.19	4.04	1.94	0.47	2.72	0.007	1.20	3.12
**Washing hands before meals**												
Never/rarely	1.00											
Sometimes/often/always	1.13	0.55	0.26	0.798	0.44	2.94						
**Washing hands after toilet**												
Never/rarely	1.00											
Sometimes/often/always	0.81	0.56	-0.30	0.766	0.21	3.16						
**Washing hands with soap**												
Never/rarely	1.00											
Sometimes/often/always	3.06	2.19	1.56	0.119	0.75	12.47						

**Friendships**												
no friends	1.00											
1 or more friend	0.71	0.31	-0.79	0.429	0.30	1.67						
**Peer support**												
Never/rarely/sometimes	1.00											
Often/always	1.11	0.29	0.41	0.683	0.67	1.86						
**Parental involvement in school**												
Never/rarely/sometimes	1.00											
Often/always	0.72	0.19	-1.24	0.217	0.43	1.21						
**Parental understanding**												
Never/rarely/sometimes	1.00											
Often/always	1.19	0.34	0.59	0.557	0.67	2.09						
**Parental bonding**												
Never/rarely/sometimes	1.00											
Often/always	0.95	0.18	-0.28	0.777	0.66	1.37						

Table S14: Multivariable logistic regression model for adolescent stunting in India, GSHS India 2007 dataset.

	**Stunting (height-for-age <2 SD) FULL MODEL**						**Stunting (height-for-age <2 SD) REDUCED MODEL**					
	**OR**	**Bootstrap std.err.**	**z**	**p**	**95% conf. interval**		**OR**	**Bootstrap std.err.**	**z**	**p**	**95% conf. interval**	
**Age**												

Supplementary material

12- 13 years	1.00						1.00					
14 - 15 years	1.34	0.09	4.17	0.000	1.17	1.53	1.34	0.09	4.22	0.000	1.17	1.54
**Sex**												
Boy	1.00						1.00					
Girl	1.33	0.25	1.50	0.134	0.92	1.93	1.39	0.27	1.69	0.091	0.95	2.03
**5 fruits and vegs**												
<5 per day	1.00											
5 or more per day	0.93	0.15	-0.46	0.646	0.67	1.28						
**Loneliness**												
Never/rarely/sometimes	1.00											
Often/always	0.83	0.24	-0.67	0.505	0.47	1.45						
**Anxiety**												
Never/rarely/sometimes	1.00											
Often/always	0.83	0.19	-0.83	0.409	0.53	1.29						
**Tobacco**												
0 days	1.00											
1 or more days	1.11	0.53	0.23	0.822	0.44	2.83						
**Physically activity**												
<7 days per week	1.00											
7 days per week	0.84	0.12	-1.23	0.218	0.63	1.11						
**Active transportation**												
<3 days per week	1.00						1.00					

3 or more days per week	1.53	0.17	3.76	0.000	1.23	1.91	1.58	0.16	4.53	0.000	1.29	1.92
**Sedentary behaviour**												
<3 hours per day	1.00						1.00					
3 or more hours per day	0.83	0.08	-1.85	0.064	0.68	1.01	0.77	0.07	-2.72	0.007	0.64	0.93
**Tooth brushing**												
<2 times per day	1.00											
2 times or more per day	0.89	0.10	-1.00	0.316	0.72	1.11						
**Washing hands before meals**												
Never/rarely	1.00											
Sometimes/often/always	0.78	0.20	-0.99	0.324	0.47	1.28						
**Washing hands after toilet**												
Never/rarely	1.00											
Sometimes/often/always	0.86	0.25	-0.50	0.615	0.49	1.53						
**Washing hands with soap**												
Never/rarely	1.00											
Sometimes/often/always	1.11	0.16	0.68	0.494	0.83	1.47						
**Friendships**												
no friends	1.00											
1 or more friend	0.90	0.19	-0.52	0.603	0.59	1.36						
**Peer support**												
Never/rarely/sometimes	1.00											
Often/always	0.94	0.13	-0.41	0.681	0.72	1.24						

**Parental involvement in school**												
Never/rarely/sometimes	1.00											
Often/always	1.36	0.22	1.88	0.061	0.99	1.88						
**Parental understanding**												
Never/rarely/sometimes	1.00											
Often/always	1.11	0.13	0.86	0.388	0.88	1.41						
**Parental bonding**												
Never/rarely/sometimes	1.00						1.00					
Often/always	0.74	0.08	-2.87	0.004	0.61	0.91	0.83	0.09	-1.75	0.080	0.67	1.02

Table S15: Multivariable logistic regression model for adolescent thinness in India, GSHS India 2007 dataset.

	**Thinness (BMI-for-age <2 SD) FULL MODEL**						**Thinness (BMI-for-age <2 SD) REDUCED MODEL**					
	**OR**	**Bootstrap std.err.**	**z**	**p**	**95% conf. interval**		**OR**	**Bootstrap std.err.**	**z**	**p**	**95% conf. interval**	
**Age**												
12- 13 years	1.00						1.00					
14 - 15 years	1.10	0.06	1.72	0.086	0.99	1.22	1.09	0.05	1.90	0.057	1.00	1.20
**Sex**												
Boy	1.00						1.00					
Girl	0.86	0.11	-1.16	0.248	0.67	1.11	0.86	0.11	-1.10	0.270	0.67	1.12
**5 fruits and vegs**												

<5 per day	1.00											
5 or more per day	0.90	0.10	-1.03	0.303	0.73	1.10						
**Loneliness**												
Never/rarely/sometimes	1.00											
Often/always	1.08	0.21	0.39	0.697	0.74	1.57						
**Anxiety**												
Never/rarely/sometimes	1.00											
Often/always	0.78	0.17	-1.10	0.271	0.51	1.21						
**Tobacco**												
0 days	1.00											
1 or more days	0.72	0.31	-0.77	0.440	0.31	1.65						
**Physically activity**												
<7 days per week	1.00											
7 days per week	0.91	0.09	-0.97	0.330	0.76	1.10						
**Active transportation**												
<3 days per week	1.00						1.00					
3 or more days per week	1.21	0.14	1.67	0.095	0.97	1.52	1.24	0.13	2.04	0.041	1.01	1.53
**Sedentary behaviour**												
<3 hours per day	1.00						1.00					
3 or more hours per day	0.81	0.10	-1.73	0.084	0.64	1.03	0.79	0.08	-2.23	0.026	0.64	0.97
**Tooth brushing**												
<2 times per day	1.00											

2 times or more per day	0.99	0.09	-0.14	0.888	0.83	1.18						
**Washing hands before meals**												
Never/rarely	1.00											
Sometimes/often/always	0.98	0.21	-0.11	0.908	0.64	1.50						
**Washing hands after toilet**												
Never/rarely	1.00											
Sometimes/often/always	1.06	0.55	0.11	0.916	0.38	2.95						
**Washing hands with soap**												
Never/rarely	1.00											
Sometimes/often/always	0.83	0.13	-1.19	0.234	0.61	1.13						
**Friendships**												
no friends	1.00											
1 or more friend	0.88	0.11	-0.98	0.325	0.69	1.13						
**Peer support**												
Never/rarely/sometimes	1.00						1.00					
Often/always	0.73	0.08	-2.81	0.005	0.58	0.91	0.71	0.07	-3.26	0.001	0.57	0.87
**Parental involvement in school**												
Never/rarely/sometimes	1.00											
Often/always	1.03	0.13	0.22	0.824	0.80	1.33						
**Parental understanding**												
Never/rarely/sometimes	1.00											
Often/always	1.03	0.10	0.28	0.778	0.85	1.24						

**Parental bonding**												
Never/rarely/sometimes	1.00											
Often/always	0.95	0.11	-0.43	0.667	0.75	1.20						

Table S16: Multivariable logistic regression model for adolescent overweight in India, GSHS India 2007 dataset.

	Overweight (BMI-for-age >1 SD) FULL MODEL						Overweight (BMI-for-age >1 SD) REDUCED MODEL					
	**OR**	**Bootstrap std.err.**	**z**	**p**	**95% conf. interval**		**OR**	**Bootstrap std.err.**	**z**	**p**	**95% conf. interval**	
**Age**												
12- 13 years	1.00						1.00					
14 - 15 years	0.84	0.05	-3.14	0.002	0.76	0.94	0.83	0.05	-3.45	0.001	0.74	0.92
**Sex**												
Boy	1.00						1.00					
Girl	0.76	0.09	-2.33	0.020	0.60	0.96	0.73	0.08	-2.84	0.005	0.58	0.91
**5 fruits and vegs**												
<5 per day	1.00											
5 or more per day	1.20	0.17	1.33	0.183	0.92	1.58						
**Loneliness**												
Never/rarely/sometimes	1.00											
Often/always	1.37	0.21	2.06	0.039	1.02	1.84						
**Anxiety**												

Never/rarely/sometimes	1.00						
Often/always	0.75	0.17	-1.27	0.206	0.48	1.17	
**Tobacco**							
0 days	1.00						
1 or more days	0.90	0.45	-0.21	0.835	0.34	2.40	
**Physically activity**							
<7 days per week	1.00						
7 days per week	1.15	0.18	0.92	0.360	0.85	1.55	
**Active transportation**							
<3 days per week	1.00						
3 or more days per week	0.87	0.12	-1.03	0.304	0.67	1.14	
**Sedentary behaviour**							
<3 hours per day	1.00						
3 or more hours per day	1.11	0.13	0.88	0.378	0.88	1.38	
**Tooth brushing**							
<2 times per day	1.00						
2 times or more per day	0.96	0.09	-0.38	0.705	0.80	1.16	
**Washing hands before meals**							
Never/rarely	1.00						
Sometimes/often/always	1.06	0.25	0.25	0.804	0.66	1.70	
**Washing hands after toilet**							
Never/rarely	1.00						

Sometimes/often/always	0.88	0.22	-0.51	0.613	0.54	1.44	
**Washing hands with soap**							
Never/rarely	1.00						
Sometimes/often/always	1.15	0.20	0.76	0.447	0.81	1.63	
**Friendships**							
no friends	1.00						
1 or more friend	0.93	0.20	-0.32	0.751	0.61	1.42	
**Peer support**							
Never/rarely/sometimes	1.00						
Often/always	0.96	0.09	-0.44	0.663	0.79	1.16	
**Parental involvement in school**							
Never/rarely/sometimes	1.00						
Often/always	0.94	0.10	-0.56	0.572	0.76	1.16	
**Parental understanding**							
Never/rarely/sometimes	1.00						
Often/always	0.90	0.13	-0.77	0.438	0.68	1.18	
**Parental bonding**							
Never/rarely/sometimes	1.00						
Often/always	1.13	0.12	1.15	0.249	0.92	1.39	

Table S17: Multivariable logistic regression model for adolescent stunting in Maldives, GSHS Maldives 2009 dataset.

Supplementary material

	Stunting (height-for-age <2 SD) FULL MODEL						Stunting (height-for-age <2 SD) REDUCED MODEL					
	OR	Bootstrap std.err.	z	p	95% conf. interval		OR	Bootstrap std.err.	z	p	95% conf. interval	
**Age**												
12- 13 years	1.00						1.00					
14 - 15 years	1.73	0.19	4.92	0.000	1.39	2.15	1.67	0.23	3.72	0.000	1.28	2.20
**Sex**												
Boy	1.00						1.00					
Girl	1.66	0.53	1.59	0.113	0.89	3.09	1.48	0.46	1.27	0.206	0.81	2.72
**5 fruits and vegs**												
<5 per day	1.00											
5 or more per day	1.01	0.38	0.02	0.987	0.48	2.11						
**Loneliness**												
Never/rarely/sometimes	1.00											
Often/always	1.07	0.25	0.29	0.774	0.68	1.68						
**Anxiety**												
Never/rarely/sometimes	1.00											
Often/always	0.93	0.31	-0.21	0.835	0.48	1.81						
**Tobacco**												
0 days	1.00											
1 or more days	1.21	0.59	0.39	0.700	0.46	3.17						

Supplementary material

**Physically activity**						
<7 days per week	1.00					
7 days per week	0.54	0.21	-1.55	0.120	0.25	1.18
**Active transportation**						
<3 days per week	1.00					
3 or more days per week	1.16	0.27	0.63	0.529	0.74	1.81
**Sedentary behaviour**						
<3 hours per day	1.00					
3 or more hours per day	1.31	0.29	1.19	0.233	0.84	2.02
**Tooth brushing**						
<2 times per day	1.00					
2 times or more per day	1.25	0.38	0.72	0.470	0.68	2.28
**Washing hands before meals**						
Never/rarely	1.00					
Sometimes/often/always	0.81	0.56	-0.30	0.761	0.21	3.12
**Washing hands after toilet**						
Never/rarely	1.00					
Sometimes/often/always	0.65	0.69	-0.41	0.681	0.08	5.16
**Washing hands with soap**						
Never/rarely	1.00					
Sometimes/often/always	0.99	0.61	-0.02	0.983	0.29	3.34
**Friendships**						

no friends	1.00											
1 or more friend	1.15	0.65	0.26	0.799	0.39	3.45						
**Peer support**												
Never/rarely/sometimes	1.00						1.00					
Often/always	0.67	0.16	-1.72	0.086	0.42	1.06	0.60	0.12	-2.55	0.011	0.40	0.89
**Parental involvement in school**												
Never/rarely/sometimes	1.00											
Often/always	1.02	0.27	0.08	0.936	0.61	1.72						
**Parental understanding**												
Never/rarely/sometimes	1.00											
Often/always	0.94	0.25	-0.24	0.810	0.55	1.59						
**Parental bonding**												
Never/rarely/sometimes	1.00											
Often/always	1.16	0.25	0.69	0.489	0.76	1.78						

Table S18: Multivariable logistic regression model for adolescent thinness in Maldives, GSHS Maldives 2009 dataset.

	**Thinness (BMI-for-age <2 SD) FULL MODEL**						**Thinness (BMI-for-age <2 SD) REDUCED MODEL**					
	**OR**	**Bootstrap std.err.**	**z**	**p**	**95% conf. interval**		**OR**	**Bootstrap std.err.**	**z**	**p**	**95% conf. interval**	
**Age**												
12- 13 years	1.00						1.00					

14 - 15 years	1.32	0.17	2.14	0.032	1.02	1.70	1.31	0.14	2.53	0.011	1.063	1.619
**Sex**												
Boy	1.00						1.00					
Girl	0.99	0.24	-0.03	0.980	0.61	1.61	1.20	0.27	0.82	0.411	0.774	1.869
**5 fruits and vegs**												
<5 per day	1.00											
5 or more per day	0.71	0.36	-0.66	0.508	0.26	1.94						
**Loneliness**												
Never/rarely/sometimes	1.00											
Often/always	0.96	0.35	-0.10	0.917	0.47	1.97						
**Anxiety**												
Never/rarely/sometimes	1.00											
Often/always	0.99	0.29	-0.04	0.970	0.56	1.75						
**Tobacco**												
0 days	1.00											
1 or more days	1.18	0.58	0.34	0.736	0.45	3.08						
**Physically activity**												
<7 days per week	1.00											
7 days per week	0.88	0.21	-0.52	0.600	0.55	1.42						
**Active transportation**												
<3 days per week	1.00											
3 or more days per week	0.75	0.18	-1.24	0.215	0.47	1.18						

Supplementary material

**Sedentary behaviour**												
<3 hours per day	1.00						1.00					
3 or more hours per day	0.66	0.13	-2.10	0.036	0.44	0.97	0.90	0.16	-0.62	0.537	0.635	1.267
**Tooth brushing**												
<2 times per day	1.00											
2 times or more per day	1.32	0.34	1.06	0.287	0.79	2.19						
**Washing hands before meals**												
Never/rarely	1.00											
Sometimes/often/always	1.04	0.34	0.13	0.894	0.55	1.98						
**Washing hands after toilet**												
Never/rarely	1.00											
Sometimes/often/always	0.37	0.26	-1.40	0.162	0.09	1.49						
**Washing hands with soap**												
Never/rarely	1.00											
Sometimes/often/always	2.62	1.65	1.53	0.127	0.76	9.00						
**Friendships**												
no friends	1.00											
1 or more friend	0.73	0.21	-1.07	0.284	0.41	1.30						
**Peer support**												
Never/rarely/sometimes	1.00											
Often/always	1.46	0.38	1.47	0.142	0.88	2.43						
**Parental involvement in school**												

Never/rarely/sometimes	1.00						1.00					
Often/always	0.66	0.12	-2.25	0.024	0.46	0.95	0.80	0.11	-1.64	0.101	0.612	1.044
**Parental understanding**												
Never/rarely/sometimes	1.00											
Often/always	0.77	0.22	-0.91	0.361	0.44	1.34						
**Parental bonding**												
Never/rarely/sometimes	1.00											
Often/always	1.15	0.29	0.57	0.566	0.71	1.89						

Table S19: Multivariable logistic regression model for adolescent overweight in Maldives, GSHS Maldives 2009 dataset.

	**Overweight (BMI-for-age >1 SD) FULL MODEL**						**Overweight (BMI-for-age >1 SD) REDUCED MODEL**					
	**OR**	**Bootstrap std.err.**	**z**	**p**	**95% conf. interval**		**OR**	**Bootstrap std.err.**	**z**	**p**	**95% conf. interval**	
**Age**												
12- 13 years	1.00						1.00					
14 - 15 years	0.68	0.10	-2.69	0.007	0.51	0.90	0.72	0.11	-2.24	0.025	0.53	0.96
**Sex**												
Boy	1.00						1.00					
Girl	0.44	0.14	-2.50	0.012	0.23	0.84	0.47	0.16	-2.27	0.023	0.25	0.90
**5 fruits and vegs**												
<5 per day	1.00											

5 or more per day	0.75	0.31	-0.69	0.490	0.33	1.71	
**Loneliness**							
Never/rarely/sometimes	1.00						
Often/always	1.09	0.42	0.24	0.814	0.52	2.30	
**Anxiety**							
Never/rarely/sometimes	1.00						
Often/always	1.08	0.38	0.21	0.835	0.54	2.17	
**Tobacco**							
0 days	1.00						
1 or more days	0.54	0.47	-0.71	0.477	0.10	2.92	
**Physically activity**							
<7 days per week	1.00						
7 days per week	0.86	0.20	-0.62	0.533	0.55	1.37	
**Active transportation**							
<3 days per week	1.00						
3 or more days per week	0.89	0.16	-0.64	0.524	0.62	1.28	
**Sedentary behaviour**							
<3 hours per day	1.00						
3 or more hours per day	1.18	0.29	0.69	0.491	0.73	1.91	
**Tooth brushing**							
<2 times per day	1.00						
2 times or more per day	0.74	0.22	-1.03	0.301	0.42	1.31	

Supplementary material

**Washing hands before meals**												
Never/rarely	1.00											
Sometimes/often/always	1.17	0.57	0.33	0.745	0.45	3.06						
**Washing hands after toilet**												
Never/rarely	1.00											
Sometimes/often/always	1.29	0.62	0.53	0.595	0.51	3.29						
**Washing hands with soap**												
Never/rarely	1.00											
Sometimes/often/always	1.06	0.41	0.15	0.884	0.49	2.27						
**Friendships**												
no friends	1.00											
1 or more friend	1.42	0.72	0.68	0.495	0.52	3.84						
**Peer support**												
Never/rarely/sometimes	1.00											
Often/always	0.99	0.28	-0.02	0.982	0.57	1.73						
**Parental involvement in school**												
Never/rarely/sometimes	1.00						1.00					
Often/always	1.59	0.32	2.29	0.022	1.07	2.36	1.61	0.32	2.40	0.017	1.09	2.39
**Parental understanding**												
Never/rarely/sometimes	1.00											
Often/always	1.13	0.27	0.52	0.602	0.71	1.81						
**Parental bonding**												

Never/rarely/sometimes	1.00											
Often/always	0.89	0.27	-0.38	0.704	0.50	1.60						

Table S20: Multivariable logistic regression model for adolescent stunting in Nepal, GSHS Nepal 2015 dataset.

	Stunting (height-for-age <2 SD) FULL MODEL						Stunting (height-for-age <2 SD) REDUCED MODEL					
	**OR**	**Bootstrap std.err.**	**z**	**p**	**95% conf. interval**		**OR**	**Bootstrap std.err.**	**z**	**p**	**95% conf. interval**	
**Age**												
12- 13 years	1.00						1.00					
14 - 15 years	1.24	0.05	5.18	0.000	1.14	1.34	1.25	0.04	6.55	0.000	1.17	1.34
**Sex**												
Boy	1.00						1.00					
Girl	0.96	0.06	-0.69	0.491	0.85	1.08	0.96	0.07	-0.51	0.609	0.83	1.12
**5 fruits and vegs**												
<5 per day	1.00											
5 or more per day	1.02	0.27	0.07	0.948	0.61	1.71						
**Loneliness**												
Never/rarely/sometimes	1.00											
Often/always	1.65	0.41	2.00	0.045	1.01	2.68						
**Anxiety**												
Never/rarely/sometimes	1.00											

Often/always	0.77	0.17	-1.17	0.243	0.50	1.19						
**Tobacco**												
0 days	1.00											
1 or more days	0.65	0.20	-1.41	0.158	0.36	1.18						
**Physically activity**												
<7 days per week	1.00											
7 days per week	0.71	0.15	-1.58	0.113	0.47	1.08						
**Active transportation**												
<3 days per week	1.00						1.00					
3 or more days per week	0.79	0.12	-1.60	0.110	0.59	1.06	0.69	0.11	-2.31	0.021	0.51	0.95
**Sedentary behaviour**												
<3 hours per day	1.00											
3 or more hours per day	0.76	0.17	-1.24	0.216	0.49	1.18						
**Tooth brushing**												
<2 times per day	1.00											
2 times or more per day	0.96	0.10	-0.37	0.714	0.79	1.18						
**Washing hands before meals**												
Never/rarely	1.00											
Sometimes/often/always	1.64	0.83	0.97	0.333	0.60	4.43						
**Washing hands after toilet**												
Never/rarely	1.00											
Sometimes/often/always	1.28	0.53	0.60	0.546	0.57	2.87						

**Washing hands with soap**												
Never/rarely	1.00						1.00					
Sometimes/often/always	0.45	0.11	-3.34	0.001	0.28	0.72	0.66	0.14	-2.00	0.046	0.44	0.99
**Friendships**												
no friends	1.00											
1 or more friend	0.91	0.18	-0.47	0.641	0.62	1.35						
**Peer support**												
Never/rarely/sometimes	1.00											
Often/always	1.10	0.11	0.95	0.342	0.90	1.35						
**Parental involvement in school**												
Never/rarely/sometimes	1.00											
Often/always	1.00	0.12	0.03	0.972	0.79	1.27						
**Parental understanding**												
Never/rarely/sometimes	1.00											
Often/always	0.89	0.09	-1.19	0.232	0.73	1.08						
**Parental bonding**												
Never/rarely/sometimes	1.00											
Often/always	1.04	0.11	0.36	0.720	0.84	1.28						

Table S21: Multivariable logistic regression model for adolescent thinness in Nepal, GSHS Nepal 2015 dataset.

	Thinness (BMI-for-age <2 SD) FULL MODEL	Thinness (BMI-for-age <2 SD) REDUCED MODEL

Supplementary material

	OR	Bootstrap std.err.	z	p	95% conf. interval		OR	Bootstrap std.err.	z	p	95% conf. interval	
**Age**												
12- 13 years	1.00						1.00					
14 - 15 years	1.14	0.10	1.53	0.127	0.96	1.35	1.14	0.08	1.85	0.064	0.99	1.32
**Sex**												
Boy	1.00						1.00					
Girl	0.74	0.14	-1.64	0.100	0.51	1.06	0.69	0.12	-2.19	0.028	0.50	0.96
**5 fruits and vegs**												
<5 per day	1.00											
5 or more per day	1.60	0.42	1.80	0.072	0.96	2.66						
**Loneliness**												
Never/rarely/sometimes	1.00											
Often/always	0.64	0.30	-0.94	0.347	0.26	1.61						
**Anxiety**												
Never/rarely/sometimes	1.00											
Often/always	0.77	0.37	-0.54	0.590	0.30	1.99						
**Tobacco**												
0 days	1.00											
1 or more days	1.25	0.43	0.65	0.518	0.64	2.43						
**Physically activity**												
<7 days per week	1.00											

Supplementary material

7 days per week	1.10	0.24	0.43	0.666	0.72	1.68						
**Active transportation**												
<3 days per week	1.00											
3 or more days per week	1.27	0.22	1.37	0.170	0.90	1.77						
**Sedentary behaviour**												
<3 hours per day	1.00											
3 or more hours per day	1.06	0.25	0.26	0.794	0.67	1.68						
**Tooth brushing**												
<2 times per day	1.00											
2 times or more per day	0.83	0.12	-1.36	0.175	0.63	1.09						
**Washing hands before meals**												
Never/rarely	1.00											
Sometimes/often/always	0.89	0.29	-0.35	0.726	0.47	1.70						
**Washing hands after toilet**												
Never/rarely	1.00						1.00					
Sometimes/often/always	0.66	0.23	-1.19	0.235	0.33	1.31	0.63	0.20	-1.46	0.144	0.34	1.17
**Washing hands with soap**												
Never/rarely	1.00											
Sometimes/often/always	1.21	0.49	0.46	0.642	0.54	2.68						
**Friendships**												
no friends	1.00											
1 or more friend	0.61	0.36	-0.84	0.400	0.20	1.91						

**Peer support**												
Never/rarely/sometimes	1.00											
Often/always	0.94	0.10	-0.54	0.588	0.76	1.17						
**Parental involvement in school**												
Never/rarely/sometimes	1.00											
Often/always	1.18	0.20	0.98	0.326	0.85	1.65						
**Parental understanding**												
Never/rarely/sometimes	1.00											
Often/always	0.95	0.20	-0.27	0.789	0.62	1.43						
**Parental bonding**												
Never/rarely/sometimes	1.00											
Often/always	0.92	0.13	-0.58	0.559	0.69	1.22						

Table S22: Multivariable logistic regression model for adolescent overweight in Nepal, GSHS Nepal 2015 dataset.

	**Overweight (BMI-for-age >1 SD) FULL MODEL**						**Overweight (BMI-for-age >1 SD) REDUCED MODEL**					
	**OR**	**Bootstrap std.err.**	**z**	**p**	**95% conf. interval**		**OR**	**Bootstrap std.err.**	**z**	**p**	**95% conf. interval**	
**Age**												
12- 13 years	1.00						1.00					
14 - 15 years	0.65	0.05	-5.89	0.000	0.56	0.75	0.67	0.05	-4.90	0.000	0.58	0.79
**Sex**												

Supplementary material

Boy	1.00						1.00					
Girl	0.59	0.12	-2.60	0.009	0.40	0.88	0.65	0.11	-2.57	0.010	0.47	0.90
**5 fruits and vegs**												
<5 per day	1.00											
5 or more per day	0.54	0.24	-1.38	0.169	0.22	1.30						
**Loneliness**												
Never/rarely/sometimes	1.00											
Often/always	0.67	0.54	-0.49	0.622	0.14	3.25						
**Anxiety**												
Never/rarely/sometimes	1.00											
Often/always	0.80	0.50	-0.36	0.720	0.23	2.75						
**Tobacco**												
0 days	1.00											
1 or more days	0.90	0.28	-0.32	0.745	0.49	1.66						
**Physically activity**												
<7 days per week	1.00											
7 days per week	1.15	0.40	0.40	0.692	0.58	2.26						
**Active transportation**												
<3 days per week	1.00											
3 or more days per week	1.48	0.29	1.98	0.048	1.00	2.18						
**Sedentary behaviour**												
<3 hours per day	1.00											

3 or more hours per day	0.95	0.37	-0.15	0.884	0.44	2.02						
**Tooth brushing**												
<2 times per day	1.00											
2 times or more per day	1.14	0.19	0.81	0.416	0.83	1.58						
**Washing hands before meals**												
Never/rarely	1.00											
Sometimes/often/always	2.28	1.50	1.25	0.210	0.63	8.26						
**Washing hands after toilet**												
Never/rarely	1.00											
Sometimes/often/always	1.25	0.98	0.29	0.772	0.27	5.81						
**Washing hands with soap**												
Never/rarely	1.00											
Sometimes/often/always	2.16	1.74	0.96	0.338	0.45	10.48						
**Friendships**												
no friends	1.00											
1 or more friend	0.65	0.33	-0.84	0.401	0.24	1.77						
**Peer support**												
Never/rarely/sometimes	1.00											
Often/always	1.25	0.24	1.17	0.241	0.86	1.81						
**Parental involvement in school**												
Never/rarely/sometimes	1.00						1.00					
Often/always	0.59	0.12	-2.50	0.012	0.39	0.89	0.67	0.14	-1.99	0.046	0.45	0.99

**Parental understanding**												
Never/rarely/sometimes	1.00											
Often/always	1.06	0.25	0.23	0.816	0.66	1.68						
**Parental bonding**												
Never/rarely/sometimes	1.00											
Often/always	0.82	0.17	-0.96	0.336	0.54	1.23						

Table S23: Multivariable logistic regression model for adolescent stunting in Sri Lanka, GSHS Sri Lanka 2016 dataset.

	Stunting (height-for-age <2 SD) FULL MODEL						Stunting (height-for-age <2 SD) REDUCED MODEL					
	**OR**	**Bootstrap std.err.**	**z**	**p**	**95% conf. interval**		**OR**	**Bootstrap std.err.**	**z**	**p**	**95% conf. interval**	
**Age**												
12- 13 years	1.00						1.00					
14 - 15 years	0.83	0.09	-1.75	0.081	0.67	1.02	0.84	0.09	-1.64	0.101	0.68	1.03
**Sex**												
Boy	1.00						1.00					
Girl	0.97	0.19	-0.14	0.890	0.66	1.44	0.97	0.21	-0.16	0.875	0.63	1.48
**5 fruits and vegs**												
<5 per day	1.00						1.00					
5 or more per day	1.46	0.31	1.77	0.076	0.96	2.21	1.56	0.29	2.34	0.019	1.07	2.26
**Loneliness**												

Never/rarely/sometimes	1.00											
Often/always	0.65	0.27	-1.01	0.310	0.29	1.49						
**Anxiety**												
Never/rarely/sometimes	1.00											
Often/always	1.38	0.62	0.71	0.475	0.57	3.33						
**Tobacco**												
0 days	1.00						1.00					
1 or more days	0.18	0.11	-2.88	0.004	0.05	0.58	0.23	0.12	-2.86	0.004	0.08	0.63
**Physically activity**												
<7 days per week	1.00											
7 days per week	0.76	0.16	-1.34	0.179	0.51	1.14						
**Active transportation**												
<3 days per week	1.00											
3 or more days per week	1.26	0.23	1.28	0.202	0.88	1.81						
**Sedentary behaviour**												
<3 hours per day	1.00											
3 or more hours per day	0.79	0.18	-1.07	0.283	0.51	1.22						
**Tooth brushing**												
<2 times per day	1.00											
2 times or more per day	1.00	0.17	0.02	0.984	0.73	1.39						
**Washing hands before meals**												
Never/rarely	1.00											

Supplementary material

Sometimes/often/always	1.00	0.77	-0.01	0.995	0.22	4.52						
**Washing hands after toilet**												
Never/rarely	1.00											
Sometimes/often/always	0.72	0.54	-0.44	0.657	0.16	3.14						
**Washing hands with soap**												
Never/rarely	1.00											
Sometimes/often/always	0.64	0.22	-1.28	0.202	0.32	1.27						
**Friendships**												
no friends	1.00						1.00					
1 or more friend	0.61	0.16	-1.92	0.055	0.37	1.01	0.61	0.12	-2.42	0.015	0.41	0.91
**Peer support**												
Never/rarely/sometimes	1.00											
Often/always	1.17	0.16	1.11	0.267	0.89	1.54						
**Parental involvement in school**												
Never/rarely/sometimes	1.00											
Often/always	1.25	0.28	1.01	0.312	0.81	1.92						
**Parental understanding**												
Never/rarely/sometimes	1.00						1.00					
Often/always	0.72	0.12	-1.96	0.049	0.52	1.00	0.78	0.08	-2.40	0.017	0.64	0.96
**Parental bonding**												
Never/rarely/sometimes	1.00											
Often/always	0.74	0.14	-1.63	0.103	0.51	1.06						

Table S24: Multivariable logistic regression model for adolescent thinness in Sri Lanka, GSHS Sri Lanka 2016 dataset.

	Thinness (BMI-for-age <2 SD) FULL MODEL						Thinness (BMI-for-age <2 SD) REDUCED MODEL					
	OR	Bootstrap std.err.	z	p	95% conf. interval		OR	Bootstrap std.err.	z	p	95% conf. interval	
**Age**												
12- 13 years	1.00						1.00					
14 - 15 years	1.04	0.10	0.40	0.689	0.86	1.25	1.03	0.09	0.39	0.698	0.88	1.22
**Sex**												
Boy	1.00						1.00					
Girl	0.86	0.17	-0.75	0.450	0.58	1.28	0.86	0.14	-0.93	0.351	0.62	1.19
**5 fruits and vegs**												
<5 per day	1.00						1.00					
5 or more per day	0.79	0.11	-1.69	0.092	0.60	1.04	0.78	0.11	-1.72	0.085	0.58	1.04
**Loneliness**												
Never/rarely/sometimes	1.00											
Often/always	1.51	0.47	1.34	0.181	0.83	2.76						
**Anxiety**												
Never/rarely/sometimes	1.00											
Often/always	0.71	0.50	-0.48	0.629	0.18	2.79						
**Tobacco**												

0 days	1.00						
1 or more days	1.75	1.28	0.77	0.441	0.42	7.34	
**Physically activity**							
<7 days per week	1.00						
7 days per week	1.19	0.32	0.64	0.520	0.70	2.00	
**Active transportation**							
<3 days per week	1.00						
3 or more days per week	1.08	0.22	0.37	0.715	0.72	1.60	
**Sedentary behaviour**							
<3 hours per day	1.00						
3 or more hours per day	0.78	0.15	-1.26	0.207	0.53	1.15	
**Tooth brushing**							
<2 times per day	1.00						
2 times or more per day	1.12	0.27	0.47	0.639	0.70	1.78	
**Washing hands before meals**							
Never/rarely	1.00						
Sometimes/often/always	0.41	0.19	-1.91	0.056	0.17	1.02	
**Washing hands after toilet**							
Never/rarely	1.00						
Sometimes/often/always	3.28	2.54	1.53	0.126	0.72	14.98	
**Washing hands with soap**							
Never/rarely	1.00						

Sometimes/often/always	1.15	0.62	0.26	0.793	0.40	3.28	
**Friendships**							
no friends	1.00						
1 or more friend	0.60	0.24	-1.29	0.196	0.28	1.30	
**Peer support**							
Never/rarely/sometimes	1.00						
Often/always	0.88	0.16	-0.71	0.480	0.62	1.25	
**Parental involvement in school**							
Never/rarely/sometimes	1.00						
Often/always	0.84	0.19	-0.81	0.419	0.54	1.29	
**Parental understanding**							
Never/rarely/sometimes	1.00						
Often/always	1.05	0.19	0.24	0.808	0.73	1.50	
**Parental bonding**							
Never/rarely/sometimes	1.00						
Often/always	0.92	0.22	-0.34	0.733	0.58	1.47	

Table S25: Multivariable logistic regression model for adolescent overweight in Sri Lanka, GSHS Sri Lanka 2016 dataset.

	**Overweight (BMI-for-age >1 SD) FULL MODEL**						**Overweight (BMI-for-age >1 SD) REDUCED MODEL**					
	**OR**	**Bootstrap std.err.**	**z**	**p**	**95% conf. interval**		**OR**	**Bootstrap std.err.**	**z**	**p**	**95% conf. interval**	

Supplementary material

**Age**												
12- 13 years	1.00						1.00					
14 - 15 years	0.91	0.11	-0.78	0.435	0.72	1.15	0.91	0.12	-0.72	0.472	0.70	1.18
**Sex**												
Boy	1.00						1.00					
Girl	0.91	0.19	-0.45	0.650	0.61	1.37	0.86	0.17	-0.74	0.458	0.59	1.27
**5 fruits and vegs**												
<5 per day	1.00											
5 or more per day	1.23	0.27	0.93	0.353	0.80	1.88						
**Loneliness**												
Never/rarely/sometimes	1.00											
Often/always	1.19	0.55	0.38	0.707	0.48	2.93						
**Anxiety**												
Never/rarely/sometimes	1.00											
Often/always	0.80	0.45	-0.40	0.688	0.27	2.39						
**Tobacco**												
0 days	1.00											
1 or more days	1.97	1.64	0.82	0.412	0.39	10.01						
**Physically activity**												
<7 days per week	1.00						1.00					
7 days per week	0.41	0.13	-2.88	0.004	0.23	0.76	0.40	0.11	-3.29	0.001	0.23	0.69
**Active transportation**												

<3 days per week	1.00											
3 or more days per week	0.73	0.18	-1.24	0.216	0.45	1.20						
**Sedentary behaviour**												
<3 hours per day	1.00						1.00					
3 or more hours per day	1.70	0.36	2.52	0.012	1.12	2.56	1.56	0.31	2.24	0.025	1.06	2.30
**Tooth brushing**												
<2 times per day	1.00											
2 times or more per day	0.98	0.23	-0.07	0.941	0.62	1.56						
**Washing hands before meals**												
Never/rarely	1.00											
Sometimes/often/always	2.57	2.11	1.15	0.251	0.51	12.84						
**Washing hands after toilet**												
Never/rarely	1.00											
Sometimes/often/always	0.44	0.30	-1.20	0.232	0.11	1.69						
**Washing hands with soap**												
Never/rarely	1.00											
Sometimes/often/always	1.08	0.59	0.13	0.895	0.37	3.15						
**Friendships**												
no friends	1.00											
1 or more friend	0.82	0.35	-0.47	0.639	0.35	1.91						
**Peer support**												
Never/rarely/sometimes	1.00											

Often/always	0.99	0.17	-0.06	0.948	0.70	1.40						
**Parental involvement in school**												
Never/rarely/sometimes	1.00						1.00					
Often/always	1.44	0.40	1.30	0.193	0.83	2.50	1.50	0.38	1.63	0.103	0.92	2.45
**Parental understanding**												
Never/rarely/sometimes	1.00											
Often/always	0.77	0.17	-1.20	0.229	0.50	1.18						
**Parental bonding**												
Never/rarely/sometimes	1.00											
Often/always	1.15	0.33	0.48	0.633	0.65	2.01						

Table S26: Multivariable logistic regression model for adolescent stunting in Bhutan, GSHS Bhutan 2016 dataset.

	**Stunting (height-for-age <2 SD) FULL MODEL**						**Stunting (height-for-age <2 SD) REDUCED MODEL**					
	**OR**	**Bootstrap std.err.**	**z**	**p**	**95% conf. interval**		**OR**	**Bootstrap std.err.**	**z**	**p**	**95% conf. interval**	
**Age**												
12- 13 years	1.00						1.00					
14 - 15 years	0.83	0.09	-1.75	0.081	0.67	1.02	0.84	0.09	-1.64	0.101	0.68	1.03
**Sex**												
Boy	1.00						1.00					
Girl	0.97	0.19	-0.14	0.890	0.66	1.44	0.97	0.21	-0.16	0.875	0.63	1.48

Supplementary material

**5 fruits and vegs**												
<5 per day	1.00						1.00					
5 or more per day	1.46	0.31	1.77	0.076	0.96	2.21	1.56	0.29	2.34	0.019	1.07	2.26
**Loneliness**												
Never/rarely/sometimes	1.00											
Often/always	0.65	0.27	-1.01	0.310	0.29	1.49						
**Anxiety**												
Never/rarely/sometimes	1.00											
Often/always	1.38	0.62	0.71	0.475	0.57	3.33						
**Tobacco**												
0 days	1.00						1.00					
1 or more days	0.18	0.11	-2.88	0.004	0.05	0.58	0.23	0.12	-2.86	0.004	0.08	0.63
**Physically activity**												
<7 days per week	1.00											
7 days per week	0.76	0.16	-1.34	0.179	0.51	1.14						
**Active transportation**												
<3 days per week	1.00											
3 or more days per week	1.26	0.23	1.28	0.202	0.88	1.81						
**Sedentary behaviour**												
<3 hours per day	1.00											
3 or more hours per day	0.79	0.18	-1.07	0.283	0.51	1.22						
**Tooth brushing**												

<2 times per day	1.00											
2 times or more per day	1.00	0.17	0.02	0.984	0.73	1.39						
**Washing hands before meals**												
Never/rarely	1.00											
Sometimes/often/always	1.00	0.77	-0.01	0.995	0.22	4.52						
**Washing hands after toilet**												
Never/rarely	1.00											
Sometimes/often/always	0.72	0.54	-0.44	0.657	0.16	3.14						
**Washing hands with soap**												
Never/rarely	1.00											
Sometimes/often/always	0.64	0.22	-1.28	0.202	0.32	1.27						
**Friendships**												
no friends	1.00						1.00					
1 or more friend	0.61	0.16	-1.92	0.055	0.37	1.01	0.61	0.12	-2.42	0.015	0.41	0.91
**Peer support**												
Never/rarely/sometimes	1.00											
Often/always	1.17	0.16	1.11	0.267	0.89	1.54						
**Parental involvement in school**												
Never/rarely/sometimes	1.00											
Often/always	1.25	0.28	1.01	0.312	0.81	1.92						
**Parental understanding**												
Never/rarely/sometimes	1.00						1.00					

Often/always	0.72	0.12	-1.96	0.049	0.52	1.00	0.78	0.08	-2.40	0.017	0.64	0.96
**Parental bonding**												
Never/rarely/sometimes	1.00											
Often/always	0.74	0.14	-1.63	0.103	0.51	1.06						

Table S27: Multivariable logistic regression model for adolescent thinness in Bhutan, GSHS Bhutan 2016 dataset.

	**Thinness (BMI-for-age <2 SD) FULL MODEL**						**Thinness (BMI-for-age <2 SD) REDUCED MODEL**					
	**OR**	**Bootstrap std.err.**	**z**	**p**	**95% conf. interval**		**OR**	**Bootstrap std.err.**	**z**	**p**	**95% conf. interval**	
**Age**												
12- 13 years	1.00						1.00					
14 - 15 years	1.04	0.10	0.40	0.689	0.86	1.25	1.04	0.09	0.43	0.665	0.88	1.22
**Sex**												
Boy	1.00						1.00					
Girl	0.86	0.17	-0.75	0.450	0.58	1.28	0.89	0.15	-0.66	0.508	0.63	1.25
**5 fruits and vegs**												
<5 per day	1.00						1.00					
5 or more per day	0.79	0.11	-1.69	0.092	0.60	1.04	0.78	0.11	-1.68	0.094	0.59	1.04
**Loneliness**												
Never/rarely/sometimes	1.00											
Often/always	1.51	0.47	1.34	0.181	0.83	2.76						

**Anxiety**												
Never/rarely/sometimes	1.00											
Often/always	0.71	0.50	-0.48	0.629	0.18	2.79						
**Tobacco**												
0 days	1.00											
1 or more days	1.75	1.28	0.77	0.441	0.42	7.34						
**Physically activity**												
<7 days per week	1.00											
7 days per week	1.19	0.32	0.64	0.520	0.70	2.00						
**Active transportation**												
<3 days per week	1.00											
3 or more days per week	1.08	0.22	0.37	0.715	0.72	1.60						
**Sedentary behaviour**												
<3 hours per day	1.00											
3 or more hours per day	0.78	0.15	-1.26	0.207	0.53	1.15						
**Tooth brushing**												
<2 times per day	1.00											
2 times or more per day	1.12	0.27	0.47	0.639	0.70	1.78						
**Washing hands before meals**												
Never/rarely	1.00						1.00					
Sometimes/often/always	0.41	0.19	-1.91	0.056	0.17	1.02	0.63	0.29	-1.01	0.311	0.25	1.55
**Washing hands after toilet**												

Never/rarely	1.00						
Sometimes/often/always	3.28	2.54	1.53	0.126	0.72	14.98	
**Washing hands with soap**							
Never/rarely	1.00						
Sometimes/often/always	1.15	0.62	0.26	0.793	0.40	3.28	
**Friendships**							
no friends	1.00						
1 or more friend	0.60	0.24	-1.29	0.196	0.28	1.30	
**Peer support**							
Never/rarely/sometimes	1.00						
Often/always	0.88	0.16	-0.71	0.480	0.62	1.25	
**Parental involvement in school**							
Never/rarely/sometimes	1.00						
Often/always	0.84	0.19	-0.81	0.419	0.54	1.29	
**Parental understanding**							
Never/rarely/sometimes	1.00						
Often/always	1.05	0.19	0.24	0.808	0.73	1.50	
**Parental bonding**							
Never/rarely/sometimes	1.00						
Often/always	0.92	0.22	-0.34	0.733	0.58	1.47	

Table S28: Multivariable logistic regression model for adolescent overweight in Bhutan, GSHS Bhutan 2016 dataset.

Supplementary material

	Overweight (BMI-for-age >1 SD) FULL MODEL						Overweight (BMI-for-age >1 SD) REDUCED MODEL					
	**OR**	**Bootstrap std.err.**	**z**	**p**	**95% conf. interval**		**OR**	**Bootstrap std.err.**	**z**	**p**	**95% conf. interval**	
**Age**												
12- 13 years	1.00						1.00					
14 - 15 years	0.91	0.11	-0.78	0.435	0.72	1.15	0.91	0.12	-0.70	0.486	0.71	1.18
**Sex**												
Boy	1.00						1.00					
Girl	0.91	0.19	-0.45	0.650	0.61	1.37	0.89	0.17	-0.63	0.526	0.61	1.28
**5 fruits and vegs**												
<5 per day	1.00											
5 or more per day	1.23	0.27	0.93	0.353	0.80	1.88						
**Loneliness**												
Never/rarely/sometimes	1.00											
Often/always	1.19	0.55	0.38	0.707	0.48	2.93						
**Anxiety**												
Never/rarely/sometimes	1.00											
Often/always	0.80	0.45	-0.40	0.688	0.27	2.39						
**Tobacco**												
0 days	1.00											
1 or more days	1.97	1.64	0.82	0.412	0.39	10.01						

Supplementary material

**Physically activity**												
<7 days per week	1.00						1.00					
7 days per week	0.41	0.13	-2.88	0.004	0.23	0.76	0.40	0.11	-3.30	0.001	0.23	0.69
**Active transportation**												
<3 days per week	1.00											
3 or more days per week	0.73	0.18	-1.24	0.216	0.45	1.20						
**Sedentary behaviour**												
<3 hours per day	1.00						1.00					
3 or more hours per day	1.70	0.36	2.52	0.012	1.12	2.56	1.46	0.30	1.82	0.068	0.97	2.19
**Tooth brushing**												
<2 times per day	1.00											
2 times or more per day	0.98	0.23	-0.07	0.941	0.62	1.56						
**Washing hands before meals**												
Never/rarely	1.00											
Sometimes/often/always	2.57	2.11	1.15	0.251	0.51	12.84						
**Washing hands after toilet**												
Never/rarely	1.00											
Sometimes/often/always	0.44	0.30	-1.20	0.232	0.11	1.69						
**Washing hands with soap**												
Never/rarely	1.00											
Sometimes/often/always	1.08	0.59	0.13	0.895	0.37	3.15						
**Friendships**												

no friends	1.00						
1 or more friend	0.82	0.35	-0.47	0.639	0.35	1.91	
**Peer support**							
Never/rarely/sometimes	1.00						
Often/always	0.99	0.17	-0.06	0.948	0.70	1.40	
**Parental involvement in school**							
Never/rarely/sometimes	1.00						
Often/always	1.44	0.40	1.30	0.193	0.83	2.50	
**Parental understanding**							
Never/rarely/sometimes	1.00						
Often/always	0.77	0.17	-1.20	0.229	0.50	1.18	
**Parental bonding**							
Never/rarely/sometimes	1.00						
Often/always	1.15	0.33	0.48	0.633	0.65	2.01	

Supplementary material
